# Analyzing Anisotropic Exchange in a Pentanuclear Os_2_Ni_3_ Complex

**DOI:** 10.1002/chem.202101972

**Published:** 2021-08-18

**Authors:** Andreas Heimermann, Christoph van Wüllen

**Affiliations:** ^1^ Fachbereich Chemie and Forschungszentrum OPTIMAS Technische Universität Kaiserslautern 67663 Kaiserslautern Germany

**Keywords:** ab initio calculations, anisotropic exchange, magnetic properties, negative g value, osmium

## Abstract

Spin Hamiltonian parameters of a pentanuclear Os${}_{2}^{\mathrm{III}}$ Ni${}_{3}^{\mathrm{II}}$ cyanometallate complex are derived from ab initio wave function based calculations, namely valence‐type configuration interaction calculations with a complete active space including spin‐orbit interaction (CASOCI) in a single‐step procedure. While fits of experimental data performed so far could reproduce the data but the resulting parameters were not satisfactory, the parameters derived in the present work reproduce experimental data and at the same time have a reasonable size. The one‐centre parameters (local g matrices and single‐ion zero field splitting tensors) are within an expected range, the anisotropic exchange parameters obtained in this work for an Os−Ni pair are not exceedingly large but determine the low‐*T* part of the experimental *χT* curve. Exchange interactions (both isotropic and anisotropic) obtained from CASOCI have to be scaled by a factor of 2.5 to obtain agreement with experiment, a known deficiency of such types of calculation. After scaling the parameters, the isotropic Os−Ni exchange coupling constant is J=-4.2 cm^−1^ and the *D* parameter of the (nearly axial) anisotropic Os−Ni exchange is $D=J_{∥}-J_{⊥}=18.8\;\;cm$ ^−1^, so anisotropic exchange is larger in absolute size than isotropic exchange. The negative value of the isotropic *J* (indicating antiferromagnetic coupling) seemingly contradicts the large‐temperature behaviour of the temperature dependent susceptibility curve, but this is caused by the negative *g* value of the Os centres. This negative *g* value is a universal feature of a pseudo‐octahedral coordination with $t_{2g}^{5}$ configuration and strong spin‐orbit interaction. Knowing the size of these exchange interactions is important because Os(CN)${}_{6}^{3-}$ is a versatile building block for the synthesis of 5d /3d magnetic materials.

## Graphical abstract

**Magnetic properties** of a pentanuclear Os${}_{2}^{\mathrm{III}}$ Ni${}_{3}^{\mathrm{II}}$ complex have been calculated from complete active space wave function calculations including spin‐orbit coupling. The calculated anisotropic Os−Ni exchange interactions in combination with a negative *g* value for octahedral Os^III^ centres reproduce the experimentally observed temperature dependence of the magnetic susceptibility.

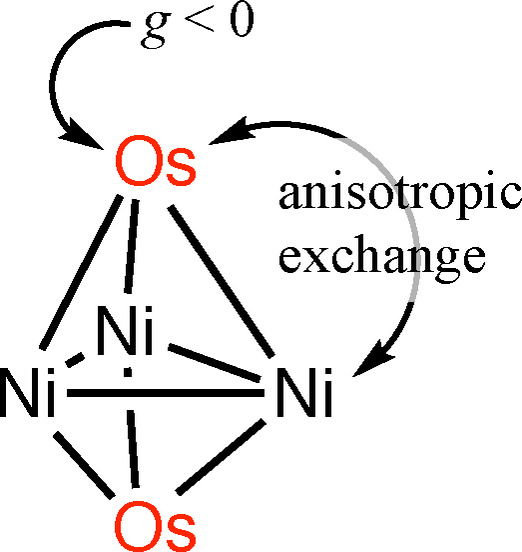

## Introduction

Open‐shell transition metal centres usually have localised unpaired electrons which are the carriers of their electronic and magnetic properties. Compared to the (naked) ions, the electronic and magnetic structure gets more involved for transition metal centres stabilised by diamagnetic ligands because of ligand‐field splittings, that is, the symmetry reduction caused by the ligand field. Still, the magnetic properties of single centres remain somewhat boring, since there is a limited range of possibilities what they can do. But as soon as several such centres interact with each other, the richness of the electronic and magnetic structure grows very strongly, in principle exponentially with the number of interacting transition metal centres. Since this often leads to novel properties, one can say that there is some sort of cooperation between the metal centres to create these properties. Possible applications are rather diverse, and different applications require distinctly different magnetic properties. For example, for magnetic storage^[1]^ it is required that a sample, once magnetised by an external magnetic field, keeps the magnetisation as long as possible after the field has been removed, which usually requires a well‐separated ground state with large magnetic anisotropy. In magnetic cooling^[2]^ on the other hand, the magnetised sample must, when removing the magnetic field, efficiently convert thermal energy into demagnetisation, which requires a high density of states and no sizeable magnetic anisotropy. The notion of what is a „good“ or „bad“ magnetic behaviour, and whether the interaction between the transition metal centres is termed „cooperative“ or „destructive“, thus largely depends on the beholder. What remains universally true is that one has to analyse and understand the properties of the building blocks and in particular their interactions before any attempt at synthesising targets with specific properties can be made.

An important step towards understanding the complex behaviour of oligonuclear transition metal complexes is to reproduce their physical behaviour in a typical „magnetic“ experiment (where only energy levels close to the ground state are populated) by a phenomenological spin Hamiltonian.[^3^, ^4^] The mathematical form of the (many‐) spin Hamiltonian used in this work is

(1) $$\hat{H}=-\sum_{i<j}J^{\left(ij\right)}{\vec{s}}^{\left(i\right)}\cdot {\vec{s}}^{\left(j\right)}+\mu_{B}\sum_{i}\vec{B}\cdot g^{\left(i\right)}\cdot {\vec{s}}^{\left(i\right)}+\sum_{i}{\vec{s}}^{\left(i\right)}\cdot D^{\left(i\right)}\cdot {\vec{s}}^{\left(i\right)}\;\;-\sum_{i<j}{\vec{s}}^{\left(i\right)}\cdot D^{\left(ij\right)}\cdot {\vec{s}}^{\left(j\right)}$$

where the $g^{\left(i\right)}$ matrices describe local magnetic moments of spin centre *i* and the single‐ion zero field splitting (ZFS) $D^{\left(i\right)}$ tensors describe the interaction of this local spin with its environment. These parameters are associated with a certain spin centre *i* and are often transferable between spin centres of the same type in a similar environment. The isotropic exchange coupling constants $J^{\left(ij\right)}$ in the first term, and the anisotropic exchange tensors $D^{\left(ij\right)}$ in the last term describe the (isotropic and anisotropic) interaction between centres *i* and *j*, the $D^{\left(ij\right)}$ are trace‐less (any trace can be absorbed in the $J^{\left(ij\right)}$ ), and they can also be assumed symmetric, any anti‐symmetric part parametrises the Dzyaloshinskii‐Moriya (DM) interaction[^5^, ^6^] which we do not consider explicitly in this work: In many cases, the DM interaction only has a small impact on the magnetic properties at least in the strong exchange limit, where the isotropic exchange dominates antisymmetric exchange.

In transition metal complexes, spin‐orbit coupling is the major microscopic origin of the g matrix anisotropy and of the anisotropic exchange interactions, much more important than direct spin‐spin interactions. Therefore, oligonuclear aggregates having both 3d and 5d transition metal centres are very interesting, since both types of spin centres contribute very different properties. 3d centres often have several unpaired electrons and thus can make a large contribution to the overall magnetic moment of the aggregate. On the other hand, the high nuclear charge of the 5d centres leads to large spin‐orbit coupling constants and thus fosters magnetic anisotropy.^[7]^ The combination of such types of centres opens a chemical space where interesting magnetic properties can emerge. There is, however, still a gap in understanding such aggregates: the physics behind single‐centre g matrices and D tensors is not so difficult to understand, and these quantities are also routinely obtained experimentally from mononuclear species. Both from experiment and theory, the understanding of isotropic exchange coupling constants $J^{\left(ij\right)}$ is also well developed.^[8]^ This is, however, much less the case for anisotropic exchange interactions. For example, these interactions are often not included when experimentalists model the measured magnetic properties of their substances with a spin Hamiltonian. For example, Hilfiger et al.^[9]^ reported the synthesis and magnetic properties of a new pentanuclear cluster with two Os^III^ and three Ni^II^ centres. While the magnetic properties could be reproduced with a spin Hamiltonian having only isotropic exchange interactions, this fit led to Ni^II^ single‐ion D tensors that were „unrealistically large“ (citation from Ref. [9]), and the authors attributed this to the fact that anisotropic exchange was not considered when fitting the magnetic data. In a subsequent publication^[10]^ the same group presented another fit of the same experimental data, where an anisotropic exchange interaction between Os^III^ and Ni^II^ was considered but no single‐ion ZFS at the Ni^II^ centres. A certain amount of temperature‐independent paramagnetism had to be assumed in this fit to achieve agreement between the experimental and fitted *χT* curve in the range 150–300 K. Using exchange interactions with [Os^III^(CN)_6_]^3–^ to boost the blocking temperature of single‐molecule magnets has further been investigated experimentally^[11]^. Quite generally, to obtain a fit that both reproduces experimental data and provides meaningful fitting parameters, one often has to impose constraints on the fit parameters based on prior experience, but the „knowledge base“ for anisotropic exchange still seems somewhat limited. Therefore the purpose of the present work is to calculate anisotropic exchange in the pentanuclear Os_2_Ni_3_ cluster of Ref. [9] based on ab initio all‐electron wave functions and then to set up a spin Hamiltonian whose parameters are extracted from such calculations. If magnetic properties (such as the magnetic susceptibility as a function of the temperature, or the magnetisation as a function of the magnetic field) calculated with such a spin Hamiltonian reproduce experimental data, confidence in the physical significance of the spin Hamiltonian parameters is greatly increased.

The hexacyano osmate(III) building block opens a large chemical space when combined with 3d centres, and although this building block is isotropic in the sense that it cannot have a single‐ion D tensor, the large spin‐orbit coupling constant of the Os centres supports substantial anisotropic exchange interactions. Anisotropic exchange interactions in compounds of this type has already been discussed by Mironov,^[12]^ but the computational procedure that led to the results is not completely clear to the present author, and no calculations on existing compounds where one could directly compare with experimental data has been performed. More detailed analyses were presented for the anisotropic exchange interaction between (high‐spin) Mn^III^ and Os^III^.[^13^, ^14^] Without spin‐orbit interaction, the three (spatial) components of the ${}^{2}T_{2g}$ state that arises from the low‐spin $t_{2g}^{5}$ configuration at the Os^III^ centre have different (isotropic) exchange interactions with the 3d metal coordinated to one of the cyano ligands, depending on which of the three *t*_2*g*_ orbitals is singly occupied. For example, if the cyano ligand is oriented along the *z* axis, the *π* type interaction is strongest but only occurs if one of the *d_xz_* and *d_yz_* metal orbitals is singly occupied.^[15]^ The *δ* type interaction which occurs if the *d_xy_* orbital is singly occupied is much smaller, and this is especially true if the interaction is mediated by a cyano ligand which only has *σ* and *π* type orbitals. So in a linear Os−CN−M arrangement (with M a 3d metal), one expects that two of spatial the components of the Os ${}^{2}T_{2g}$ have a stronger exchange interaction with M than the third one. These considerations apply to a situation without spin‐orbit coupling. Taking into account the strong spin‐orbit coupling at the Os atom, the six micro‐states from the ${}^{2}T_{2g}$ are strongly mixed and result in a lower Kramers doublet well separated by ∼4500 cm^−1^ from two other such doublets. Regarding the lower doublet as a pseudo spin with S=1/2 , the differences in the isotropic exchange couplings in the spin‐orbit‐free case then lead (at least phenomenologically) to an anisotropic exchange interaction.[^13^, ^14^]

The purpose of the present work is therefore to evaluate anisotropic exchange in the pentanuclear Os_2_Ni_3_ cluster of Ref. [9] based on ab initio all‐electron wave functions, and to reproduce the experimental magnetic data with a spin Hamiltonian whose parameters are derived from such calculations. In the following sections we describe our computational approach and present the results, including numerical experiments that demonstrate the relative importance of different spin Hamiltonian parameters to be able to reproduce the experimental data.

## Methods and Materials

### Molecular geometry

**Figure 1:**
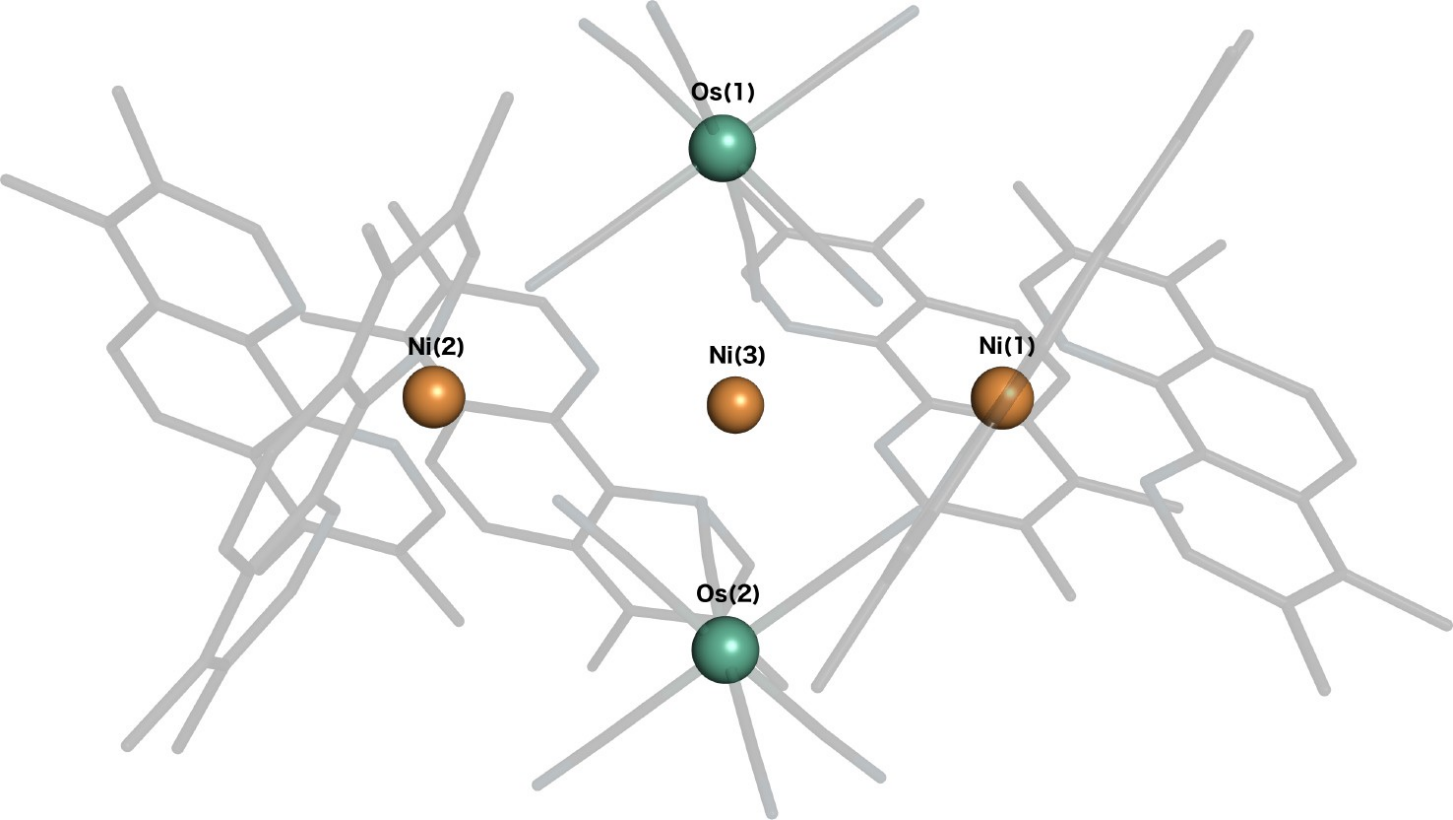
Molecular structure of the „Os_2_Ni_3_“ complex.

The pentanuclear charge‐neutral complex [Ni(tmphen)_2_]_3_[Os(CN)_6_]_2_ (tmphen=3,4,7,8‐tetramethyl‐1,10‐phenantroline) features a trigonal bipyramidal arrangement of the five metal centres (see Figure 1). The two Os atoms are the apices of the bipyramid, while the three Ni atoms are in its equatorial plane. Each Os atom is octahedrally coordinated by six cyano ligands, three of which point outwards, the other three coordinate to each of the Ni atoms. For each of the Ni atoms, the sixfold coordination is completed by two tmphen ligands. There are thus six Os‐CN−Ni bridges (from both Os to the three Ni) that promote the Os−Ni exchange interaction. The complex has been synthesised and its temperature dependent magnetic susceptibility measured in Ref. [9], from that source we obtained a molecular geometry from the X‐ray crystal structure, as well as the field dependent magnetisation data. The experimental *χT* curve has again be presented in Figure 4 of Ref. [10] which has been used as the source of experimental data to compare with. To facilitate the analysis, we slightly modified the geometry such that it adopts axial *C*_3_ symmetry. To this end, a fully diamagnetically substituted variant (all Os^III^ replaced by Ir^III^ and all Ni^II^ replaced by Zn^II^ was optimised in *C*_3_ symmetry. The geometry was optimised using density functional theory (DFT) with the PBE0 exchange‐correlation functional^[16]^ and def2‐TZVP basis sets,^[17]^ using a scalar quasi‐relativistic effective core potential replacing 60 core electrons^[18]^ at the Ir atoms. The DFT calculations were performed with a local version of the TURBOMOLE program[^19^, ^20^, ^21^] using the Berny algorithm^[22]^ as implemented in the Gaussian16 program^[23]^ for the update of the coordinates. Note that there is no horizontal mirror plane, so the two Os atoms (Os_1_ and Os_2_) are not symmetry equivalent, while the three Ni centres (Ni_3_, Ni_4_, Ni_5_) are. The tensorial parameters of the spin Hamiltonian ($g^{\left(i\right)}$ , $D^{\left(i\right)}$ and $D^{\left(ij\right)}$ ) depend on the orientation of the molecule, so following Ref. [10], an orientation was chosen where Os_1,2_ are on the positive and negative *z* axis, while Ni_3,4,5_ are in the *xy* plane, with Ni_3_ on the *x* axis. The cartesian coordinates are documented in the Supporting Information and used in all subsequent calculations.

### CASOCI wave functions

A qualitatively correct wave function for an oligonuclear transition metal complex is necessarily highly multi configurational if antiferromagnetic coupling and/or spin‐orbit interaction is important, therefore we use a complete active space (CAS) valence‐type configuration interaction (CI) wave function. The CI is performed using a new CASOCI (complete active space spin‐orbit CI) program described in detail elsewhere.^[24]^ The calculations use a quasi relativistic fourth‐order Douglas‐Kroll Hamiltonian,[^25^, ^26^] two‐electron spin‐orbit effects were including by constructing the matrix elements of a spin‐orbit mean‐field operator^[27]^ which were then used as an *input* to our Douglas‐Kroll „machine”. To extract g matrices from CASOCI wave functions, one needs the matrix elements of the Zeeman operator (see following paragraph), and we used the non‐relativistic Zeeman operator ($\hat{L}+g_{e}\hat{S}$ ), with *g_e_* the free‐electron *g* value, for that purpose.

The orbitals used in the CASOCI calculation were obtained from scalar‐relativistic restricted open‐shell Hartree‐Fock (ROHF) calculations using the scalar‐relativistic part of the Douglas‐Kroll operator. In the all‐electron calculations, a TZVP basis set^[28]^ was used for the non‐metal (N, C, H) atoms, while a basis sets derived from a compilation of Hirao and Nakajima^[29]^ was used for Os, Ir, Ni, and Zn. To facilitate the calculations with and without replacing Os/Ir and Ni/Zn, the basis sets of these atom pairs had the same overall structure, such that molecular orbital coefficients could be used as start orbitals across diamagnetic substitutions. For Ni and Zn, we started from the primitive 20s15p9d basis set and contracted the steepest 7 *s*, the steepest 5 *p*, and the steepest 4 *d* functions. Then, a diffuse *p* and *d* function was added, and then a set of *f* functions taken from the QZVPP^[30]^ basis. For Ir and Os, we started from the 27s23p15d10f primitive basis, contracted the steepest 10s6p5d6 f functions, and added one diffuse *p*, *d*, and *f* function. Contraction coefficients have been derived from atomic calculations with the fourth‐order Douglas‐Kroll Hamiltonian, and the all‐electron basis sets thus constructed are documented in the Supporting Information.

The details of the ROHF orbital optimisation, including the Roothaan parameters, are found in the Supporting Information. Here we just mention that the problem with optimising the scalar‐relativistic orbitals comes from those either empty (Os *e_g_*) or fully occupied (Ni *t*_2*g*_) in the high‐spin ROHF reference configuration(s). Empty orbitals are not optimised at all, while fully occupied orbitals can mix with the doubly occupied ligand orbitals without changing the wave function. Both problems are addressed by including Slater determinants which have local excitations, e. g. high‐spin $t_{2g}^{3}e_{g}^{2}$ at the Os^III^ atoms or $t_{2g}^{5}e_{g}^{3}$ at the Ni^II^ atoms. Since our data is extracted from calculations in which different numbers of metal centres have been „muted“ by diamagnetic substitution, it is important to impose size consistency, that is, the orbitals optimised at one centre do not depend on whether another weakly interacting centre is active or diamagnetically substituted. While this requirement is very difficult to meet when using state‐averaged CASSCF calculations to optimise the orbitals (which is a standard procedure for mononuclear complexes) it is straightforward to impose in the ROHF case (see Support Information). The ROHF energy is defined as a weighted average of the energy expectation values of the (ground and excited configuration) Slater determinants considered, and for such an energy expression the orbitals can be optimised with standard methods.

In the present case, with 34 active electrons (five from each Os^III^ and eight from each Ni^II^) in 25 orbitals (five 5d orbitals for each metal centre), this results in a CI‐dimension of $4.9{}^{*}10^{12}$ and it would at least be very difficult with a conventional CAS‐CI program. While one can come close to the full CAS‐CI solution using the density matrix renormalisation group (DMRG) approach,[^31^, ^32^] such a calculation would not necessarily produce additional insight: What interests us is how the properties of the building blocks and their interactions build the electronic and magnetic structure of the overall system. Therefore we use *diamagnetic substitution* as a technique to extract such one‐centre and two‐centre terms. Diamagnetic substitution means that individual spin centres are „muted“ by replacing them with a diamagnetic centre of the same charge and about the same size, without changing the molecular geometry. In our case, Os^III^ (low‐spin octahedral *d* ^5^) is replaced by Ir^III^ (*d* ^6^) and Ni^II^ (octahedral *d* ^8^) is replaced by Zn^II^ (*d* ^10^). Diamagnetic substitution is also used in experimental investigations and occasionally is a challenge to synthesis, but in quantum chemical calculations one can make such substitutions very easily.

### Extracting spin Hamiltonian parameters from ab initio wave functions

Effective Hamiltonian theory is the basis for connecting microscopic (ab initio) and phenomenological (spin) Hamiltonians.[^33^, ^34^, ^35^, ^36^] Since CASOCI is not a perturbative method but diagonalises the CI matrix, the model space is formed by selecting, when the CASOCI calculation has completed, some states well separated from the others. The effective (giant) spin $\vec{S}$ is then implicitly determined by the number of model space functions which amounts to 2S+1 . Then matrix elements of the Zeeman operators (for determining the $g^{\mathrm{eff}}$ matrix or matrix elements of the zero‐field Hamiltonian (for determining ZFS tensor $D^{\mathrm{eff}}$ ) are mapped onto the matrix elements of an effective single‐spin Hamiltonian of the form

(2) $$\begin{array}{c} \;{\hat{H}}_{\mathrm{eff}}=\mu_{B}\vec{B}\cdot g^{\mathrm{eff}}\cdot \vec{S}+\vec{S}\cdot D^{\mathrm{eff}}\cdot \vec{S} \end{array}$$

It is, however usually not possible to get a perfect match of the model space and spin Hamiltonian matrix elements. For example, the Zeeman part of the spin Hamiltonian can only have matrix elements between $\left|SM\right\rangle$ model space spin states where the values of *M* differ by 1 at most, while it is possible that the ab initio model space functions have a non‐zero matrix elements between corresponding model space functions where *M* differs by more than one. An exact match can always be found if one includes higher‐order spin operators in the spin Hamiltonian,^[35]^ but this leads to a spin Hamiltonian with a very large set of parameters. Instead, our approach is to perform a least‐squares procedure to define the lowest‐order spin Hamiltonian (Eq. 2) that gives the best match of the matrix elements.^[37]^ In cases where spin‐orbit interaction is weak, the choice of the model space basis functions is usually obvious, because the ab initio wave functions are still close to pure spin functions. One can also systematically determine the model space functions by an adiabatic connection formalism^[35]^ where one starts with ab initio wave functions obtained without spin orbit coupling (which can be chosen as pure spin functions) and then adiabatically switches on the spin‐orbit interaction and rotates the ab initio wave functions at each new (increased) value of the spin orbit coupling such that spin Hamiltonian parameters evolve continuously. In the present case, this is not possible because of the strong spin orbit interaction at the Os centre, which produces a low‐energy Kramers doublet in which all components from the ${}^{2}T_{2g}$ manifold are heavily mixed. In other words, having such a Kramers doublet it is not clear which linear combination of the two components of the doublet maps to the spin‐up and which to the spin‐down model function. To remove the ambiguity, the wave function analysis module of the CASOCI program^[24]^ follows a suggestion by Chibotaru and Unger^[38]^ which can be viewed as the choice which makes the g matrix as diagonal as possible. First one determines the magnetic axes of the model space by constructing the Abragam‐Bleaney tensor G as

(3) $$\begin{array}{c} \;G_{\kappa \lambda }=\frac{3}{S\left(S+1\right)\left(2S+1\right)}\left\langle \Psi_{i}\right.\left|{\hat{H}}_{\mathrm{Zeeman}}^{\kappa }\Psi_{j}\right\rangle \left\langle \Psi_{i}\right.\left|{\hat{H}}_{\mathrm{Zeeman}}^{\lambda }\Psi_{i}\right\rangle \end{array}$$

where the Ψ_*i*,*j*_ run over all components of the model space, κ,λ=x,y,z and ${\hat{H}}_{\mathrm{Zeeman}}^{\kappa }$ is a cartesian component of the magnetic field Zeeman operator. The important point here is the G is invariant to a unitary mixing of the Ψ_*i*_. If (and only if) there is a perfect match of the matrix elements in the model space and the matrix elements of the spin Hamiltonian, G as defined here equals $g^{\mathrm{eff}}\cdot {\left(g^{\mathrm{eff}}\right)}^{T}$ where the superscript *T* denotes taking the transpose of the matrix. Diagonalising G gives the magnetic axes, and then one diagonalises the Zeeman operator within the model space for a magnetic field along the main magnetic axis and sorts the resulting eigenvectors by the interaction energy, this gives the coefficients of the model space functions Ψ_*M*_ (which are mapped to the $\left|SM\right\rangle$ basis functions of the spin Hamiltonian) as linear combinations of the Ψ_*i*_. Of course, diagonalisation only specifies the Ψ_*M*_ up to a complex phase factor *c_M_*, and these phase factors are determined as to make the $g^{\mathrm{eff}}$ matrix as diagonal as possible. To fix the phase, we have to use all three magnetic axes that form a right‐handed coordinate system and transform the Zeeman matrix elements of the Ψ_*M*_ to this new system, where the matrix representation of ${\hat{H}}_{\mathrm{Zeeman}}^{z}$ is diagonal by construction with monotonically rising diagonal elements. The phase of the Ψ_*M*_ is then adjusted such that

(4) $$\begin{array}{c} \;{\left\{ℑ\left\langle \Psi_{M-1}\right.\left|{\hat{H}}_{\mathrm{Zeeman}}^{x}\Psi_{M}\right\rangle \right\}}^{2}+{\left\{ℜ\left\langle \Psi_{M-1}\right.\left|{\hat{H}}_{\mathrm{Zeeman}}^{Y}\Psi_{M}\right\rangle \right\}}^{2} \end{array}$$

is minimised (this makes $g_{xy}^{\mathrm{eff}}$ and $g_{yx}^{\mathrm{eff}}$ small, see Eq. (33) in Ref. [37]) and then possibly a minus sign is applied to Ψ_*M*_ to make5, 6

(5) $$\begin{array}{cc} ℜ\left\langle \Psi_{M-1}\right.\left|{\hat{H}}_{\mathrm{Zeeman}}^{x}\Psi_{M}\right\rangle & >0 \end{array}$$

(6) $$\begin{array}{cc} ℑ\left\langle \Psi_{M-1}\right.\left|{\hat{H}}_{\mathrm{Zeeman}}^{y}\Psi_{M}\right\rangle & >0 \end{array}$$

and if both conditions cannot be fulfilled simultaneously, (only) the condition involving the matrix element that is larger in absolute value is fulfilled. This convention to construct the model space functions makes $g_{zz}^{\mathrm{eff}}>0$ (because the Ψ_*M*_ are sorted by their interaction energy with the magnetic field) and the positivity conditions make at least one of *g_xx_*, *g_yy_* positive. While the sign of individual *g* values is *not* a physical observable, the sign of their product is. We use the wording „sign of the *g* value“ to indicate the sign of the product of the three *g* values. This sign determines the orientation of the precession of the effective spin around the axis of an external magnetic field,^[39]^ and matches the sign of the expression

(7) $$\frac{6}{S\left(S+1\right)\left(2S+1\right)}ℑ\sum_{i,j,k}\left\langle \Psi_{i}\right.\left|{\hat{H}}_{\mathrm{Zeeman}}^{x}\Psi_{j}\right\rangle \left\langle \Psi_{j}\right.\left|{\hat{H}}_{\mathrm{Zeeman}}^{y}\Psi_{k}\right\rangle \left\langle \Psi_{k}\right.\left|{\hat{H}}_{\mathrm{Zeeman}}^{z}\Psi_{i}\right\rangle$$

which is also invariant with respect to a unitary mixing of the Ψ_*i*_ and equals the determinant of $g^{\mathrm{eff}}$ (and therefore the product of the *g* values) if the model space and spin Hamiltonian matrix elements match. In the case of the Os^III^ centres, this expression is negative (see below), so the construction of the model space functions Ψ_*M*_ produces *g_xx_* and *g_yy_* being equal in absolute value but having different sign, which seemingly contradicts the axial symmetry of the system. This can be cured by rotating the reference frame of the spin operators in the spin Hamiltonian (see next section). The whole procedure extracts a *molecular* $g^{\mathrm{eff}}$ that refers to the effective (giant) spin of the many‐spin system, while we are mainly interested in the g matrices of the individual spin centres. Therefore we use diamagnetic substitution and in a series of calculations, all spin centres except one are „muted“, and the resulting $g^{\mathrm{eff}}$ is then the g matrix of the un‐muted centre. After having specified the model space functions, the Hamiltonian matrix which is diagonal (with the ab initio micro‐state energies as diagonal elements) in the space of the ab initio wave functions Ψ_*i*_ is transformed to the model space functions, and from that matrix representation the effective ZFS tensor $D^{\mathrm{eff}}$ is extracted. As for the $g^{\mathrm{eff}}$ matrices, we use the equations we have given in Ref. [37]. This is how the single‐ion D tensors of the Ni^II^ centres are extracted from CASOCI calculations where only one Ni centre is un‐muted.

The exchange interaction parameters for a given Os−Ni pair are extracted from CASOCI calculations where all but one Os and one Ni centre are un‐muted. The problem here is, that the effective ZFS tensor $D^{\mathrm{eff}}$ for such an Os−Ni pair will depend both on the single‐ion ZFS tensor at Ni and on the anisotropic exchange tensor of the Os−Ni pair. To disentangle these two contributions, we have performed a calculation where the spin‐orbit matrix elements for Ni‐centred orbitals were set to zero. As a result, we get three Kramers doublets, the lower two doublets energetically close and separated from the highest one. This can be reproduced by an isotropic ferromagnetic exchange interaction, with the lower quartet further split by zero field splitting. Other interpretations are possible (see next section), but this one allows to apply relations only valid in the strong exchange limit. In this limit, for two spins with $S_{1}=1/2$ and $S_{2}=3/2$ 8

(8) $$\begin{array}{c} \;D^{\mathrm{eff}}=\frac{1}{3}D^{\left(12\right)} \end{array}$$

so the anisotropic exchange tensor for a Os−Ni pair with spin‐orbit interaction switched off at the Ni centre is just three times the effective ZFS tensor for that pair. The isotropic *J* value for that pair is then simply adjusted such that the spin Hamiltonian reproduces the energy spacing to the uppermost Kramers doublet. Of course, one must then check whether the CASOCI results from a calculation where the entire spin‐orbit interaction is present are reproduced by a spin Hamiltonian where the single‐ion D tensor or the Ni centre is included, and these tests were successful (see the Supporting Information). Because of the strong spin‐orbit interaction at the Os centres and the ambiguity to define the reference frame of the spin operators there, one must now be careful to combine spin Hamiltonian parameters extracted from CASOCI calculations with different diamagnetic substitution patterns. There is an ambiguity in the choice of the spin Hamiltonian parameters and this will be spelled out in the next section.

The full tensors are given in the Supporting Information, here we give the *g* values (the eigenvalues of the single‐centre $g^{\left(i\right)}$ matrices) and the *D* and *E* parameters of the single‐ion $D^{\left(i\right)}$ and anisotropic exchange $D^{ij)}$ tensors. Since these tensors are traceless, their eigenvalues $D_{1},D_{2},D_{3}$ sum so zero, and sorting them such that *D*_3_ is the largest in absolute value, the *D* and *E* parameters are defined as

(9) $$\begin{array}{cc} D=\; & D_{3}-\frac{1}{2}\left(D_{1}+D_{2}\right)\; \end{array}$$

(10) $$\begin{array}{cc} E= & \frac{1}{2}\left|D_{1}-D_{2}\right| \end{array}$$

### Spin rotations in the spin Hamiltonian

To demonstrate the ambiguity of the spin Hamiltonian parameters, we start with the effective (single‐spin) Hamiltonian Eq. (2) and choose new spin operators ${\tilde{S}}_{\kappa }$ which arise from rotating the original ones11

(11) $$\begin{array}{c} \;{\tilde{S}}_{\kappa }=R_{\lambda \kappa }{\hat{S}}_{\lambda } \end{array}$$

(κ=x,y,z ). The rotation matrix R is not arbitrary. First, the new spin operators should be Hermitian which implies that R is a real matrix. Then, $S^{2}={\tilde{S}}^{2}$ should hold, from this follows that R is an orthogonal matrix. Finally, the usual angular momentum commutation relations of spin operators should also hold for the ${\tilde{S}}_{\kappa }$ which implies that the determinant of R is positive (+1). Since R is orthogonal, it is also easy to express the original spin operators through the rotated ones12

(12) $$\begin{array}{c} \;S_{\kappa }=R_{\kappa \lambda }{\tilde{S}}_{\lambda } \end{array}$$

We now want to re‐write the single‐spin Hamiltonian in terms of the new spin operators ${\tilde{S}}_{\kappa }$ and arrive at13, 14, 15

(13) $$\begin{array}{cc} {\hat{H}}^{\mathrm{eff}}\; & =\mu_{B}\vec{B}\cdot g^{\mathrm{eff}}\cdot \vec{S}+\vec{S}\cdot D^{\mathrm{eff}}\cdot \vec{S}\;\\ \; & =\mu_{B}\vec{B}\cdot {\tilde{g}}^{\mathrm{eff}}\cdot \vec{\tilde{S}}+\vec{\tilde{S}}\cdot {\tilde{D}}^{\mathrm{eff}}\cdot \vec{\tilde{S}}\; \end{array}$$

(14) $$\begin{array}{cc} {\tilde{g}}^{\mathrm{eff}} & =g^{\mathrm{eff}}\cdot R \end{array}\;$$

(15) $${\tilde{D}}^{\mathrm{eff}}=R^{T}\cdot D^{\mathrm{eff}}\cdot R$$

Note that this equation means that the two Hamiltonians (expressed through the un‐primed and primed spin operators) are the same and thus have the same matrix elements with a given set of basis functions. So two spin Hamiltonians with very different parameters reproduce the same physics, which also means that the G tensor, the product of the *g* values, and the *D* and *E* values are the same for both reference spin frames. Such a rotation of the frame of the giant (effective) spin has already been discussed in Refs. [35, 37]. In the present case, such rotations are necessary to transform a g matrix with one negative diagonal element (as obtained initially for the Os centres) to one with three negative diagonal elements which represent the symmetry of the environment of the Os centre. More involved is the case where one rotates the reference frames of two local spins w.r.t. each other in the many‐spin Hamiltonian Eq. (1). Here one rotates each spin individually, described by a rotation matrix $R^{\left(i\right)}$ for each centre16

(16) $$\begin{array}{c} \;{\tilde{s}}_{\kappa }^{\left(i\right)}=R_{\kappa \lambda }^{\left(i\right)}s_{\lambda }^{\left(i\right)} \end{array}$$

The one‐centre parameters of the many‐spin Hamiltonian transform in the same way as for the single effective spin Hamiltonian except that now each centre has its individual rotation matrix17, 18

(17) $${\tilde{g}}^{\left(i\right)}={\left(R^{\left(i\right)}\right)}^{T}\cdot g^{\left(i\right)}$$

(18) $${\tilde{D}}^{\left(i\right)}={\left(R^{\left(i\right)}\right)}^{T}\cdot D^{\left(i\right)}\cdot R^{\left(i\right)}$$

but two‐centre (exchange) interactions transform in a special way. Here one must combine the isotropic and anisotropic exchange into a single matrix $X^{\left(ij\right)}$ and then calculate its transformed form

(19) $$X^{\left(ij\right)}=J^{\left(ij\right)}I+D^{\left(ij\right)}$$

(20) $${\tilde{X}}^{\left(ij\right)}={\left(R^{\left(i\right)}\right)}^{T}\cdot X^{\left(ij\right)}\cdot R^{\left(j\right)}$$

(I is the 3×3 unit matrix), before one can determine the transformed ${\tilde{J}}^{\left(ij\right)}$ as one third of the trace of ${\tilde{X}}^{\left(ij\right)}$ and ${\tilde{D}}^{\left(ij\right)}$ as the symmetric part of ${\tilde{X}}^{\left(ij\right)}$ after making it traceless. Note further that ${\tilde{X}}^{\left(ij\right)}$ is not necessarily symmetric since the rotation matrices of two different centres have been applied. This means that if for two centres the spin reference frames are rotated differently, then this may introduce a DM interaction where there was none using the original frames. This is not surprising because the DM interaction is an energetic contribution that comes from spin canting, and whether two spins are canted or not depends on the relative orientation of their reference frame. In the same way, isotropic and anisotropic exchange is transformed into each other.

These considerations seem to be quite esoteric at first sight, but are of importance in the present context. We want to extract spin Hamiltonian parameters for the pentanuclear Os_2_Ni_3_ complex from *different* CASOCI calculations on variants where one Os and one Ni centre is un‐muted, and these parameters at the end have to fit together in a single spin Hamiltonian. The strong spin‐orbit coupling at the Os centre, together with its negative *g* value, leads to different local spin frames in different calculations. For example, we have chosen the frames in the “mono‐nuclear” calculations such that the g matrices of both *Os* and *Ni* are more or less diagonal. From this one expects for an Os−Ni pair (at least within the strong exchange limit) that the resulting molecular G matrix is diagonal as well, which was not the case for the CASOCI data. This could be rectified by assuming that the local spin frame at the Os centre was rotated, and we determined the rotation that produced the off‐diagonal G in the CASOCI calculation in the spin Hamiltonian calculation as well. Then we rotated back to the original frame which changes the exchange parameters, both $J^{\left(ij\right)}$ and $D^{\left(ij\right)}$ . As a result, we have obtained a spin Hamiltonian where the Os spin frame is in the original position *and* which produces results that match CASOCI. Note that this „back‐rotation“ also produced a small amount of DM interaction because the transformed $\tilde{X}$ matrices were not symmetric. We left out this antisymmetric part after we have verified that a spin Hamiltonian with and without the DM interaction produces essentially the same results.

## Results and Discussion

As a first step, the one‐body spin Hamiltonian parameters (local $g^{\left(i\right)}$ matrices and single‐ion $D^{\left(i\right)}$ tensors) were extracted from a set of five calculations where all but one spin centre was muted, following the procedures outlined above. This step is relatively easy since for only a single un‐muted centre, the effective (molecular) g and D tensors can be identified with those associated with the un‐muted canter. The resulting g matrices and D tensors were symmetry equivalent to a high degree of accuracy. For the use in spin Hamiltonian calculations we take the symmetrised matrices/tensors which keep the equivalency of the Ni centres in the spin Hamiltonian. The principal values of these one‐body matrices/tensors are presented in Table 1. Although the two Os centres are not strictly symmetry equivalent, their *g* values match to two decimals. The Os g matrices are diagonal and axial, with the *z* component markedly different from the *x,y* components. Most noteworthy is, however, that the Os centres have negative *g* values. While the sign of the individual *g* values is somewhat arbitrary (see paragraph on spin rotations in the Methods section), their product $g_{1}g_{2}g_{3}$ is uniquely defined and determines a physical observable, namely the orientation of the precession of the magnetic moment.^[39]^ The sign of the *g* value can be detected in spectroscopic experiments but will not affect magnetisation and magnetic susceptibility. The average Os^III^ *g* value ($g_{av}=-1.93$ ) obtained with CASOCI should be compared with $\left|g\right|=1.82$ reported from a broad EPR resonance for mono‐nuclear [Ph_4_P]_3_[Os(CN)_6_]^[40]^ (this experiment does not indicate the sign of *g*). We are not aware that negative *g* values have been discussed for Os^III^ so we investigated this using a ligand field simulation implemented as a „notebook“ for the computer algebra program mathematica.^[41]^ For an octahedral *d* ^5^ complex with an infinitely large octahedral splitting and a strong spin‐orbit coupling we find21

(21) $$\begin{array}{c} \;g=-\frac{g_{e}+4k}{3} \end{array}$$

**Table 1:** One‐centre spin Hamiltonian parameters of the Os_2_Ni_3_ complex (principal values, D and E in cm^−1^). Note the negative *g* values of the Os centres. The full tensors are given in the Supporting Information.

Centre	g matrix			D tensor	
	*g*_1_	*g*_2_	*g*_3_	*D*	*E*
Os_1_	−2.08	−2.08	−1.63		
Os_2_	−2.08	−2.08	−1.63		
Ni_3,4,5_	2.29	2.32	2.34	−4.86	1.50

where *k* is the so‐called orbital reduction factor,^[42]^ which is applied to the angular momentum matrix elements. This expression agrees with expressions given in the literature[^10^, ^13^, ^15^] except for the negative sign. The ligand field simulation shows that the negative *g* value is a universal feature of the $t_{2g}^{5}$ occupation with large spin‐orbit splitting. An orbital reduction factor of k=0.85 has been used to reproduce $\left|g\right|=1.8$ as found in EPR experiments.^[11]^ It must be noted, however, that in reality the finite value of the octahedral splitting leads to spin‐orbit induced mixing with excited quartet states, so with an octahedral splitting of $\Delta_{\mathrm{oct}}=10Dq=38500$ cm^−1^ and a spin‐orbit coupling constant of 3100 cm^−1^ as suggested in Ref. [40], the *g* value from our ligand field simulation changes from −1.8 to −1.9 (when using k=0.85 ) and this only weakly depends on the value of the Racah parameter *B*. While most experiments on a mononuclear compound are not affected by the sign of the *g* value, this is different when such a centre is exchange coupled to other spin centres.

The Ni g matrices are nearly isotropic with g≈2.3 . There is a significant orbital contribution to the Ni magnetic moments (since g>2 ), but the calculated *g* value is in the expected range (albeit at its upper end). For the Ni single‐ion D tensors we find an extreme rhombicity ($\left|E/D\right|∼\frac{1}{3}$ ), so the sign of *D* is meaningless. Such a large rhombicity means that one eigenvalue of D is close to zero, while the other two have opposite sign. The eigenvector corresponding to the near zero eigenvalue is parallel to the *z* axis (the axis parallel to a line connecting the two Os) for all three Ni centres, the other eigenvectors being in the equatorial (*xy*) plane of the bipyramid formed by the five metal centres.

Relevant exchange interactions are only expected between the Os and Ni centres, since they are connected by a cyano bridge. A CASOCI calculation on a system with two un‐muted Ni centres and with the spin‐orbit interaction switched off yields a quintet ground state, with a triplet 0.002 cm^−1^ and a singlet 0.003 cm^−1^ higher. This implies a very weak ($J_{\mathrm{NiNi}}=0.001$ cm^−1^) ferromagnetic coupling between the Ni centres, which can certainly be ignored. There are only two non‐equivalent Os−Ni pairs, and their exchange interaction parameters are listed in Table 2. The parameters as extracted from the CASOCI calculations are marked with the attribute *unscaled* (see below for the scaled parameters). Although the two Os atoms are not strictly symmetry equivalent, it can be seen that the exchange parameters are quite similar. Most striking is that the isotropic exchange coupling constants are negative (antiferromagnetic coupling). This seems to contradict the observed increase of *χT* when lowering the temperature, starting at 300 K, but the reason for this seeming discrepancy is the negative *g* value of the Os centre: a parallel spin alignment with opposite *g* values reduces the magnetic moment, compared to the uncoupled situation present at high temperatures. The anisotropic exchange tensors have quite a small rhombicity, $D^{\left(ij\right)}=7.5$ cm^−1^ together with $J^{\left(ij\right)}=-1.7$ cm^−1^ means that the exchange interaction constant is $J_{∥}=3.3$ cm^−1^ for parallel spins in one direction and $J_{⊥}=-4.2$ cm^−1^ if they are aligned perpendicular to that direction. Note that the absolute values of $J_{∥},J_{⊥}$ are not very different, and the eigenvector corresponding to the ∥ direction is also not oriented along a line connecting Os and Ni.

**Table 2:** Isotropic and anisotropic exchange interactions in the Os_2_Ni_3_ complex (principal values, in cm^–1^). Unscaled values are extracted from the CASOCI wave functions, the scaled ones are multiplied by 2.5. The full tensors are given in the Supporting Information. Ignoring the rhombicity, one can combine the *J* and *D* into $J_{∥}=J+2/3D$ , $J_{⊥}=J-1/3D$ to facilitate comparison with the literature.

Centres	*J*(ij)	*D*(ij)	*E*(ij)	$J_{∥}$ (ij)	$J_{⊥}$ (ij)
Os_1_−Ni_3_ **unscaled**	−1.69	7.50	0.70	3.31	−4.20
Os_1_−Ni_3_ **scaled**	−4.23	18.76	1.75	8.27	−10.49
Os_2_−Ni_3_ **unscaled**	−1.67	7.52	0.58	3.35	−4.18
Os_2_−Ni_3_ **scaled**	−4.17	18.81	1.45	8.37	−10.44

The microscopic origin of the anisotropic exchange is that without spin‐orbit interaction, there are very different isotropic exchange coupling constants for the states arising from the ${}^{2}T_{2g}$ manifold.[^13^, ^15^] Therefore we also looked at the scalar‐relativistic energy levels obtained in CASOCI calculations with one OsNi pair un‐muted. The energy levels are given in the Supporting Information (Table S2). We find a doublet ground state separated by ∼400 cm^−1^ from two close‐lying higher doublets. This pattern, a ^2^ *A* and a ^2^ *E* in *C*_3_ symmetry, arises from the trigonal distortion of the octahedral field at the Os centre. The small splitting ∼30 cm^−1^) calculated for the two ^2^ *E* states comes from the diamagnetic substitution pattern (one Ni^II^ and two Zn^II^) which destroys the *C*_3_ axis. For each of the three groups, exchange coupling with the Ni centre gives a pair consisting of a quartet at lower energy and a doublet slightly (2–16 cm^−1^) above, giving three isotropic positive (ferromagnetic) exchange coupling constants with 3.11, 10.76, and 1.69 cm^−1^. So the differences between these values are in the same ballpark as the anisotropy of the exchange after spin‐orbit coupling is included. To interpret the isotropic couplings, one must note that the trigonal distortion not only splits the *t*_2*g*_ orbitals but also rotates them. Taking the Ni−NC−Os axis as a the local *z* axis for the moment and considering the observed trigonal distortion of the octahedral field, one get three symmetry adapted linear combinations of the *t*_2*g*_ orbitals with22

(22) $$\begin{array}{cc} d_{1}\; & =\frac{1}{\sqrt{6}}\left(2d_{xy}-d_{xz}-d_{yz}\right)\;\\ d_{2}\; & =\frac{1}{\sqrt{2}}\left(d_{xz}-d_{yz}\right)\;\\ d_{3}\; & =\frac{1}{\sqrt{2}}\left(d_{xy}+d_{xz}+d_{yz}\right)\; \end{array}$$

where *d*_1,2_ have the same orbital energy and *d*_3_ is ∼500 cm^−1^ higher. Because of the degeneracy, there can be a unitary mixing of *d*_1_ and *d*_2_ but the choice given here corresponds to the symmetry breaking of the diamagnetic substitution. In the lowest group of the states, *d*_3_ is the magnetic orbital (singly occupied), while in the two higher groups *d*_1_ and *d*_2_ are the magnetic orbital. Since these orbitals are given in a local frame where the Os−Ni connection is the *z* axis, the *d*_*xz*,*yz*_ orbitals make the efficient (*π* type) exchange coupling while the *d_xy_*, being of *δ* type w.r.t. the Os−Ni linkage, cannot provide an efficient coupling pathway. Therefore it follows that a state with *d*_2_ singly occupied should have the largest isotropic coupling constant, while the smallest coupling constant is expected if *d*_1_ is singly occupied and an intermediate value if *d*_3_ is singly occupied. This matches the coupling constants of the scalar‐relativistic CASCI calculation (Supporting Information, Table S2) where an intermediate value (J=3.11 cm^−1^) is found for the states arising from coupling the Os ^2^ *A* state with the Ni, while the largest and the smallest value is found for the two close‐lying states arising coupling Os ^2^ *E* with the Ni. Because of the trigonal distortion of the Os coordination, the situation is therefore more complex as in simple two‐centre models (see, e. g. Ref. [13]) which have a tetragonal, corner‐shared arrangement of the octahedra of the two centres. This difference, however, is only important for the isotropic coupling of the scalar‐relativistic states. Because spin‐orbit coupling at Os is ∼10 times stronger than the trigonal distortion, the spin‐orbit induced mixing of the six micro‐states arising from the ${}^{2}T_{2g}$ manifold only weakly depends on how these states are split by the ligand field. So the argument developed in Ref.^[13]^, namely that the rhombicity of the anisotropic exchange tensor is mainly affected by the bending angle between Os‐CN and the 3d metal, remains valid. In our case, the angle is $∼160^{\circ }$ which explains the observed rhombicity ($\left|E/D\right|∼0.09$ , see Table 2) of the anisotropic exchange tensor obtained from the CASOCI calculations.

**Figure 2:**
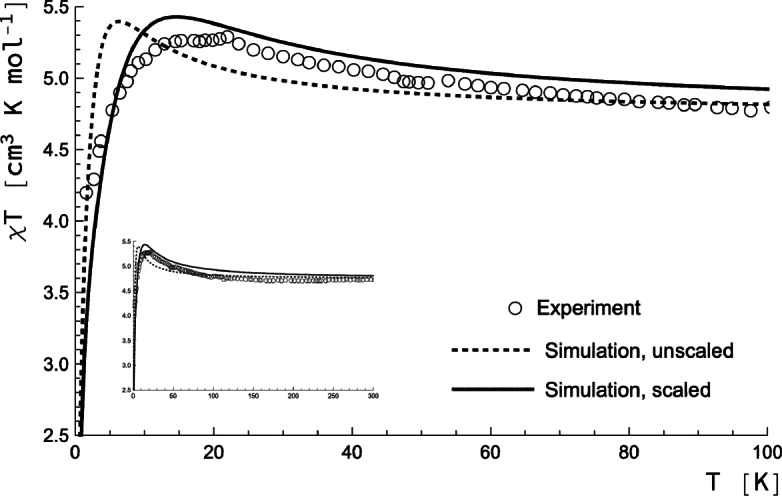
Temperature dependence of *χT* for the Os_2_Ni_3_ complex from spin Hamiltonian calculations using parameters extracted from CASOCI wave functions (solid line). The dotted („unscaled”) and solid („scaled”) have been obtained using the scaled and unscaled exchange parameters from Table 2 (see Supporting Information for the full tensors). The inset shows the curves over the full range of temperatures used in the experimental measurements. Experimental data points are from Ref. [10].

These two‐centre parameters have been validated (see the Supporting Information) by comparing spin Hamiltonian calculations on OsNiOs and NiOsNi aggregates and comparing with CASOCI calculations on the Os_2_Ni_3_ complex where two Ni centres, or one Os and one Ni centres, have been muted. With these spin Hamiltonian parameters, the temperature dependence of *χT* has been calculated (Figure 2, dotted line). The *χT* curve is only plotted up to 100 K since it gets quite boring beyond, the inset documents how the curves behave over the full temperature range. Comparing the simulated (dotted) curve with experimental data (from Ref. [10]) it can be seen that the overall behaviour of *χT* is similar to experiment, but that the peak is at much too low temperatures. This indicates that the exchange interactions are underestimated in the calculation. In fact, valence‐type CI calculations with (only) metal‐centred active orbitals are known to severely underestimate (by a factor of 2 to 3) exchange interactions (see, e. g. Refs. [36, 43]). This deficiency of CASSCF‐type calculations has been examined in detail,^[44]^ where it is attributed to the inability of the orbitals optimised for the ground state to describe charge‐transfer excitations which are important for super‐exchange. Therefore we repeated the spin Hamiltonian calculations scaling all the exchange interactions ($J^{\left(ij\right)}$ and $D^{\left(ij\right)}$ ) with 2.5 (Figure 2, solid line), and this considerably improves the agreement with experiment. The parameters which are extracted „as is“ from the CASOCI calculations are denoted *unscaled*, while the parameters obtained by multiplying these values with 2.5 have the attribute *scaled*. From the discussion of spin rotations in the Methods section, it follows that because spin rotations within the spin Hamiltonian formalism transform different types of exchange interaction into each other, the isotropic and anisotropic exchange interactions should be scaled with a common factor. While one may regard this approach as somewhat eclectic, it must be pointed out that we apply a single correction factor to address a well‐known deficiency of the computational method. So it is fair to say that the experimental *χT* curve could be reproduced using spin Hamiltonian parameters that are based on ab initio wave function based results. Of course now it is interesting to compare the anisotropic exchange parameters used here with those resulting from a fit of the experimental *χT* curve in Ref. [10]. To avoid over‐parametrisation which leads to a good fit but meaningless parameters, such a fit must restrict the number of parameters, therefore uniaxial Os−Ni anisotropic exchange tensors (with two parameters $J_{∥}$ and $J_{⊥}$ ) have been assumed, with the parallel eigenvector oriented along the Os−Ni connection in an idealised geometry. Moreover, no single‐ion ZFS tensors of the Ni^II^ centres have been included in the spin Hamiltonian when fitting the experimental data. For this reason, we repeated our simulation setting those tensors to zero as well and indeed found out that the Ni single‐ion ZFS tensors have a small influence on the simulated *χT* curve (see Supporting Information, Figure S4). The fit in Ref. [10] has $J_{∥}=47.6$ cm^−1^ (we have multiplied the value found there with 2 to compensate for an additional factor 2 in the definition of the spin Hamiltonian followed in that work) and $J_{⊥}=2.4$ cm^−1^, which are substantially different from our (scaled) values ($J_{∥}=8.3$ and $J_{⊥}=-10.4$ cm^−1^). Again, the different sign of the exchange interaction parameters follows from the fact that in Ref. [10] a positive Os *g* has been assumed but what remains is that our exchange interaction parameters are much smaller. Digging into this, we found that the exchange interaction constants of Ref. [10] are probably much too large: they produce a *χT* curve which considerably declines from 100 to 300 K although the experimental curve is essentially flat there. To compensate for this, a sizeable temperature independent paramagnetism (TIP) had to be assumed that essential „lifts“ the declining branch of the *χT* curve. Our interpretation of this is, that because of ignoring the negative Os *g* value, the exchange interactions had to be chosen that strong to reproduce the low‐T part of the *χT* curve. This led to too small susceptibilities at higher temperatures which was then compensated by adding a TIP correction, which adds a correction proportional to the temperature to the *χT* curve. Our CASOCI based (scaled) spin Hamiltonian parameters on the other hand are able to fairly reproduce the experimental data over the whole range of temperatures without including TIP in the model.

**Figure 3:**
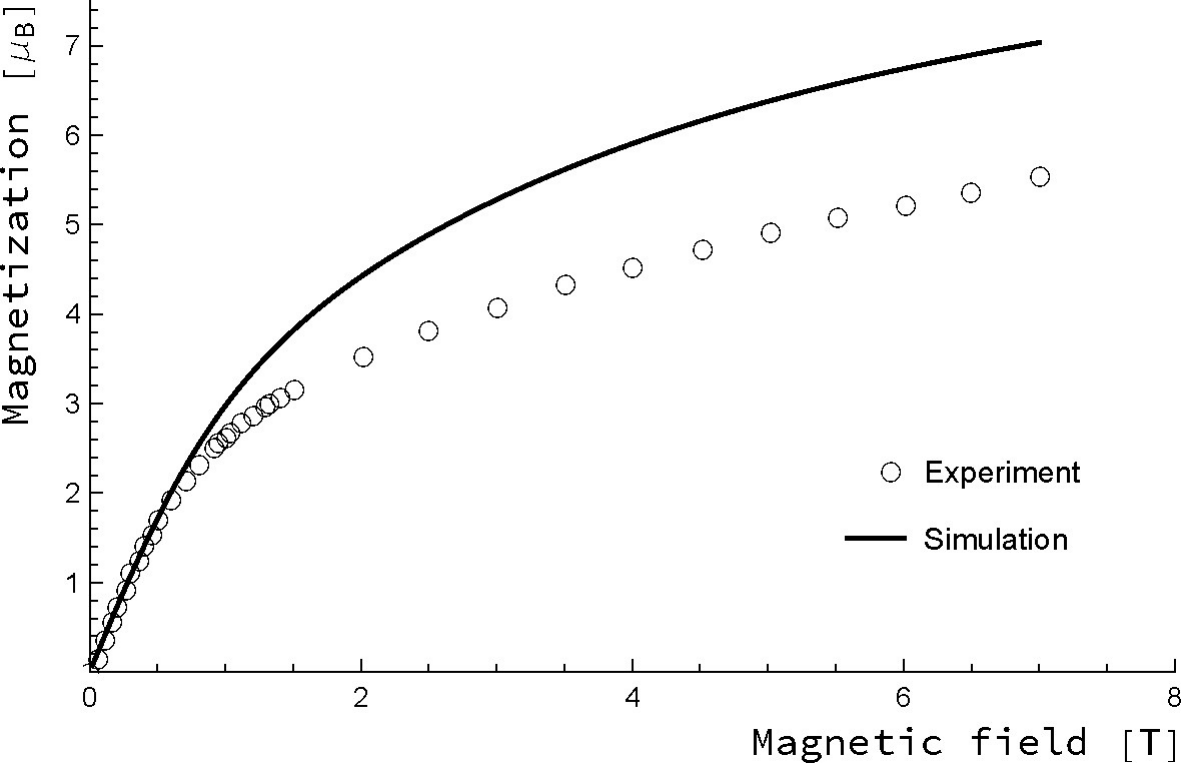
Plot of the magnetisation as a function of the magnetic field strength for a fixed temperature of 1.8 K. Scaled CASOCI parameters have been used in the simulation, experimental data points are from Ref. [9].

In order to complete the comparison of experimental data with the results of our simulation, the field‐induced magnetisation for variable magnetic fields at a fixed temperature of 1.8 K was extracted from our simulation and compared with the experimental data points from Ref. [9] (Figure 3). For small magnetic fields up to 1 T, the simulation is close to the experimental values which also implies that the magnetic susceptibility, which is the slope of that curve, is retrieved correctly. It is, however, obvious from Figure 3 that the saturation of the magnetisation experimentally observed at higher magnetic fields is not very well reproduced in the spin Hamiltonian simulation. We note in passing that this is also the case for a simulation taking the spin Hamiltonian parameters of Ref. [10]. It seems that the saturation observed both in experiment and simulation quite sensitively depends on the magnetic properties of the lowest eigenstates.

**Figure 4:**
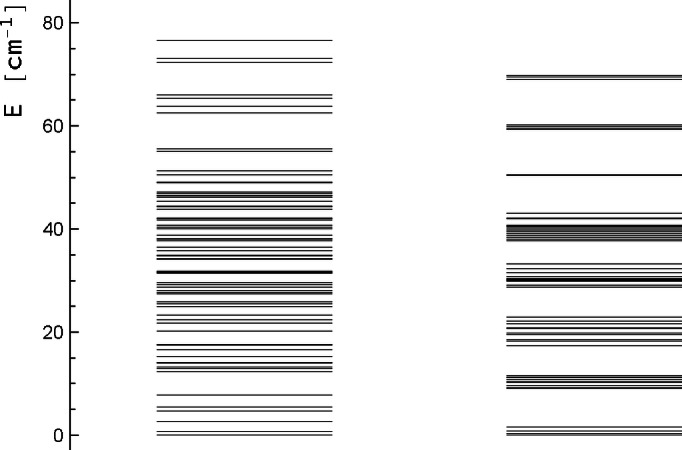
Energy of the 108 low lying micro‐states of Os_2_Ni_3_ from spin Hamiltonian calculations (scaled CASOCI parameters). Left: all spin Hamiltonian parameters included, right: Ni single‐ion D tensors set to zero.

Finally, we want to point out that although it is possible to reproduce the essential features of the experimental *χT* curve without including single‐ion ZFS tensors at the Ni^II^ centres, this does not mean that these have no influence on the magnetic properties in general. To this end, we have plotted the spectrum (108 energy levels) obtained in our simulation with and without the Ni ZFS tensors (Figure 4), using the *scaled* exchange interaction parameters since they best reproduce the experimental data. Zero field splitting at the Ni centres slightly spreads the spectrum (the left panel is more extended than the right one in Figure 4) but the main effect is that without zero field splitting at the Ni (right panel), the spectrum is much more structured, with clustered eigenvalues and gaps between the clusters. Including the Ni single‐ion ZFS tensors (left panel) fills the gaps and leads to an unstructured spectrum. This certainly adds pathways for magnetic relaxation. Fast relaxation has been observed experimentally^[9]^ (no out‐of‐phase signal down to 1.8 K in AC magnetic susceptibility measurements), and the conjecture, based on the data obtained here, is that single‐ion (Ni) zero field splitting in cooperation with anisotropic (OsNi) exchange boosts magnetic relaxation. This statement has to be taken with a grain of salt, since one must not over‐stretch the interpretation of individual spin Hamiltonian parameters.

## Conclusions

Using spin Hamiltonian parameters extracted from ab initio calculations performed on congeners of a pentanuclear Os${}_{2}^{\mathrm{III}}$ Ni${}_{3}^{\mathrm{II}}$ complex with various diamagnetic substitution patterns, it was possible to reproduce experimental magnetic properties from the spin Hamiltonian calculation. While it was necessary to scale up the exchange interactions from the underlying CASSCF‐type spin‐orbit CI calculation, the resulting spin Hamiltonian parameters look quite reasonable, and can be physically interpreted by the properties of the individual centres (g matrices and single‐ion D tensors) and their connection ($J^{\left(ij\right)}$ and $D^{\left(ij\right)}$ exchange interactions). No further corrections such as adding TIP have been necessary to fit the experimental *χT* curve over the whole temperature range. Comparing our ab initio based spin Hamiltonian parameters with those from a literature, we find marked differences. To a large part, these differences arise since a positive *g* value for the Os^III^ has seemingly been assumed so far in all fits to experimental data. While the sign of the *g* value has little effect on most experiments on mononuclear species, it strongly affects the magnetic properties once such a centre is exchange‐coupled with a „normal“ spin centre with a positive *g* value, and ignoring the negative *g* value at the Os centres when fitting experimental data naturally has a big effect on fitted exchange interaction constants. Given the richness of cyanometallate chemistry, the ability of quantifying the anisotropic exchange interaction between building blocks such as [Os(CN)_6_]^3–^ and 3d centres is of great importance for rationalising, tuning, and predicting magnetic properties in this class of oligonuclear 3d/5d compounds.

## Supporting Information

Supporting Information to this manuscript is available free of charge (PDF, 32 pages). Support Information includes the spin Hamiltonian parameters as full tensors, details on the orbital optimisation including Roothaan coefficients used and the ROHF energies for all variants obtained by different diamagnetic substitution patterns, the non‐standard basis sets for Os, Ni, Ir, and Zn for the all‐electron calculations, and the molecular geometry used.

## Conflict of interest

The authors declare no conflict of interest.

## Supporting information

As a service to our authors and readers, this journal provides supporting information supplied by the authors. Such materials are peer reviewed and may be re‐organized for online delivery, but are not copy‐edited or typeset. Technical support issues arising from supporting information (other than missing files) should be addressed to the authors.

Supporting Information
