# Supplementary material for: Analyzing Anisotropic Exchange in a Pentanuclear Os2Ni3 Complex
Source: Chemistry. 2021 Aug 18;27(61):15148–58. doi: 10.1002/chem.202101972 (PMC8597145; doi:10.1002/chem.202101972)
Supplement: Supplementary file 1 — Supporting Information [file CHEM-27-15148-s001.pdf]

Chemistry–A European Journal

Supporting Information

Analyzing Anisotropic Exchange in a Pentanuclear Os_2Ni_3 Complex

Chemistry–A European Journal

Supporting Information

Analyzing Anisotropic Exchange in a Pentanuclear Os_2Ni_3 Complex

Contents

S1 Spin Hamiltonian parameters extracted from CASOCI wave functions **S1**
S1.1 g matrices of Os_1 and Os_2 . . . . S1
S1.2 g matrices and single-ion D tensors of Ni_3, Ni_4, Ni_5 . . . . S2
S1.3 Isotropic and anisotropic exchange interaction of the OsNi pairs . S3
S1.4 Validating the spin Hamiltonian parameters S7
S1.5 Scalar-relativistic isotropic exchange in the OsNi pairs S10

S2 Effect of the Ni single-ion ZFS tensors **S10**

S3 ROHF Orbital optimisation **S12**

S4 Basis sets used for Ir, Os, Zn, Ni in TURBOMOLE format **S15**
S4.1 Ir basis S15
S4.2 Os basis S18
S4.3 Zn basis S21
S4.4 Ni basis S23

S5 Molecular geometry of the neutral ,,Os_2Ni_3" complex **S26**

S6 Restricted open shell Hartree-Fock energies **S32**

S1 Spin Hamiltonian parameters extracted from CASOCI wave functions

The parameters given here refer to a many-spin Hamiltonian as given in Eq. (1) of the manuscript and repeated here for convenience

$$\hat{H} = -\sum_{i<j} J^{(ij)} \vec{s}^{(i)} \cdot \vec{s}^{(j)} + \mu_B \sum_i \vec{B} \cdot \mathbf{g}^{(i)} \cdot \vec{s}^{(i)} + \sum_i \vec{s}^{(i)} \cdot \mathbf{D}^{(i)} \cdot \vec{s}^{(i)} - \sum_{i<j} \vec{s}^{(i)} \cdot \mathbf{D}^{(ij)} \cdot \vec{s}^{(j)} \tag{S1}$$

S1.1 g matrices of Os_1 and Os_2

These **g** matrices have been obtained from CASOCI calculations in which only one Os atom was ,,un-muted" (this means: all others were ,,muted" by a diamagnetic substitution $Os^{III} \to Ir^{III}$ and $Ni^{II} \to Zn^{II}$). It became immediately clear that the Os centres ,,have a negative g value" which means that the determinant of the **g** matrix (or the product of the three g values) is negative. From the standard phase convention used when extracting **g** matrices from CASOCI data (see Sec. 2.3 of the manuscript) the following **g** matrix (with $g_{yy} < 0$ but

$g_{xx}, g_{yy} > 0$) resulted

$$\begin{pmatrix} +2.078 & 0.000 & 0.000 \\ 0.000 & -2.077 & 0.000 \\ 0.000 & 0.000 & +1.627 \end{pmatrix} \tag{S2}$$

which does not reflect the axial symmetry of the system, which implies $g_{xx} = g_{yy}$. This is the reason why we have to perform a spin rotation (see the manuscript) that implements the substitution

$$\hat{S}_x \to -\hat{S}_x \tag{S3}$$

$$\hat{S}_y \to -\hat{S}_y \tag{S4}$$

(which is a rotation of the spin reference frame by 180° around the z axis) and finally get

$$\mathbf{g}(\mathrm{Os}_1) = \begin{pmatrix} -2.078 & 0.000 & 0.000 \\ 0.000 & -2.077 & 0.000 \\ 0.000 & 0.000 & -1.627 \end{pmatrix} \tag{S5}$$

Likewise, we obtain

$$\mathbf{g}(\mathrm{Os}_2) = \begin{pmatrix} -2.078 & 0.000 & 0.000 \\ 0.000 & -2.077 & 0.000 \\ 0.000 & 0.000 & -1.629 \end{pmatrix} \tag{S6}$$

S1.2 g matrices and single-ion D tensors of Ni_3, Ni_4, Ni_5

These parameters are extracted from CASOCI calculations where all but a single Ni centre were muted. All three CASOCI calculations feature a low-lying triplet separated by more than 6000 cm^{-1} from the other ligand-field states. In all three cases, the triplet is split such that within 0.01 cm^{-1} the second and third micro-states are 2.99 cm^{-1} and 6.35 cm^{-1} above the lowest one, which already indicates that the single-ion D tensors are quite rhombic.
The **g** matrices for the three centres in their respective magnetic axis system are diagonal and nearly isotropic with $g_1 = 2.393$, $g_2 = 2.318$ and $g_3 = 2.344$, and the magnetic z axis is oriented along the line from the origin of the molecule-fixed coordinate system to the respective Ni centre. Transformed to the molecule-fixed coordinate system, the three g matrices reflect the molecular symmetry

(one results from the others through a rotation), and the **g** matrices are

$$\mathbf{g}(\mathrm{Ni}_3) = \begin{pmatrix} 2.344 & 0.000 & -0.001 \\ 0.000 & 2.296 & 0.008 \\ -0.001 & 0.008 & 2.316 \end{pmatrix}, \tag{S7}$$

$$\mathbf{g}(\mathrm{Ni}_4) = \begin{pmatrix} 2.308 & 0.021 & 0.007 \\ 0.021 & 2.332 & -0.003 \\ 0.007 & -0.003 & 2.316 \end{pmatrix}, \tag{S8}$$

$$\mathbf{g}(\mathrm{Ni}_5) = \begin{pmatrix} 2.308 & -0.021 & -0.006 \\ -0.021 & 2.332 & -0.005 \\ -0.006 & -0.005 & 2.316 \end{pmatrix}. \tag{S9}$$

The **D** tensors for the three Ni centres were extracted from the same set of calculations. For all Ni centres, the D and E values are $D = 4.86$ cm^{-1} and $E = 2.99$ cm^{-1} (within ± 0.01 cm^{-1}), and the **D** tensors are related by C_3 rotations with very small discrepancies (~ 0.02 cm^{-1}). For the use in the Spin Hamiltonian, we have symmetrised the three tensors (such that each one exactly results from applying a rotation to the others) and get (values in cm^{-1})

$$\mathbf{D}(\mathrm{Ni}_3) = \begin{pmatrix} -3.237 & -0.001 & 0.103 \\ -0.001 & 2.861 & -0.839 \\ 0.103 & -0.839 & 0.376 \end{pmatrix}, \tag{S10}$$

$$\mathbf{D}(\mathrm{Ni}_4) = \begin{pmatrix} 1.337 & -2.640 & -0.778 \\ -2.640 & -1.713 & 0.331 \\ -0.778 & 0.331 & 0.376 \end{pmatrix}, \tag{S11}$$

$$\mathbf{D}(\mathrm{Ni}_5) = \begin{pmatrix} 1.336 & 2.641 & 0.675 \\ 2.641 & -1.712 & 0.509 \\ 0.675 & 0.509 & 0.376 \end{pmatrix}. \tag{S12}$$

Note that these tensors are not aligned along the z axis, and their tensorial sum is rather small, axial and of the hard-axis type. This is so because the easy axes for each of the three tensors is along a line from the origin of the molecule-fixed coordinate system to the respective Ni atom, that is, in the xy plane.

S1.3 Isotropic and anisotropic exchange interaction of the OsNi pairs

To extract spin Hamiltonian parameters for, say, the Os(1)Ni(3) pair, we run calculation where all spin centres except Os(1) and Ni(3) are ,,muted". As explained in the manuscript (Sec. 2.3), we first run a calculation with the spin-orbit interaction ,,switched off" at the Ni centre to extract the anisotropic exchange tensor as three times the effective (molecular) $\mathbf{D}^{\mathrm{eff}}$ tensor. The ,,raw" $\mathbf{D}^{(13)}$ tensor thus extracted reads (values in cm^{-1})

$$\begin{pmatrix} 0.6555 & 0.2901 & -0.5295 \\ 0.2901 & -0.4644 & -0.2307 \\ -0.5295 & -0.2307 & -0.1911 \end{pmatrix}$$

Table S1: CASOCI and spin Hamiltonian energies for the lowest three Kramers doublets of the complex with Os(1)Ni(3) ,,unmuted" (values in cm^{-1})

CASOCI spin-orbit OFF at Ni centre	spin Hamiltonian only OsNi anisotropic exchange	CASOCI full spin-orbit interaction	spin Hamiltonian OsNi anisotropic exchange and Ni single-ion ZFS
0.000	0.000	0.000	0.000
0.000	0.000	0.000	0.000
1.006	0.984	4.262	4.070
1.006	0.984	4.262	4.070
6.387	6.377	9.542	9.340
6.387	6.377	9.542	9.340

Calculating the spectrum of a spin Hamiltonian for two spin centres with $S_1 = \frac{1}{2}$ and $S_2 = 1$ using this anisotropic exchange tensor and an isotropic ferromagnetic exchange coupling of $J^{(13)} = +3.9$ cm^{-1} reproduces the spectrum of a CASOCI calculation in which the spin-orbit coupling is switched off at the Ni centre very well (Table S1, columns 1 and 2). To check the validity of the approach, the single-ion $\mathbf{D}$ tensor for the Ni centre as obtained in the preceding section was then included in the spin Hamiltonian and the resulting energy levels fairly match the CASOCI energy levels obtained with the full spin-orbit interaction (Table S1, columns 3 and 4).

Including the $\mathbf{g}$ matrices for Os(1) and Ni(3) as obtained before in the spin Hamiltonian produced a molecular $\mathbf{G}^{\mathrm{eff}}$ matrix that differed from the CASOCI result. We know already that we have to apply a spin rotation implementing Eq. (S3) to the Os spin frame because of the negative g value there. In addition, there must be a rotation around the spin x axis to produce a large G_{yz} element that is observed in the CASOCI calculations. Therefore a spin rotation matrix of the form

$$\mathbf{R}^{(\mathrm{Os})} = \begin{pmatrix} -1 & 0 & 0 \\ 0 & 1 & 0 \\ 0 & 0 & -1 \end{pmatrix} \begin{pmatrix} 1 & 0 & 0 \\ 0 & \cos\phi & \sin\phi \\ 0 & -\sin\phi & \cos\phi \end{pmatrix} \tag{S13}$$

was used. For a value $\phi = 0.85$ this produces, starting from the Os(1) $\mathbf{g}$ matrix given above, a matrix

$$\mathbf{g}(\mathrm{Os}) = \begin{pmatrix} 2.078 & 0.000 & 0.000 \\ 0.000 & -1.371 & -1.561 \\ 0.000 & -1.222 & 1.074 \end{pmatrix}$$

and if this ,,rotated" $\mathbf{g}$ matrix is used in the spin Hamiltonian instead of the original one, the spin Hamiltonian calculation reproduces the CASOCI data, as

demonstrated by the $\mathbf{G}$ matrices for the lowest four micro-states (effective spin $S = 3/2$):

$$\mathbf{G}^{\text{eff}}(\text{CASOCI}) = \begin{pmatrix} 5.108 & 0.033 & 0.031 \\ 0.033 & 1.110 & -1.023 \\ 0.031 & -1.023 & 2.694 \end{pmatrix}$$

$$\mathbf{G}^{\text{eff}}(\text{SpinH}) = \begin{pmatrix} 5.067 & -0.003 & -0.010 \\ -0.003 & 1.169 & -1.063 \\ -0.010 & -1.063 & 2.698 \end{pmatrix}$$

Note how the rotation of the Os frame produced the large off-diagonal yz element in the $\mathbf{G}$ matrix, and this element would be close to zero if in the spin Hamiltonian all input $\mathbf{g}$ matrices were diagonal. Now we have a spin Hamiltonian which reproduces the CASOCI data for the OsNi pair, but the Os $\mathbf{g}$ matrix does not reflect the overall molecular symmetry. Therefore one must now perform a back-rotation of the Os spin frame (see manuscript) such that the $\mathbf{g}$ matrix is rotated back to its original form while not changing the spin Hamiltonian (and leaving all observables such as the $\mathbf{G}$ matrix invariant), therefore the Os-Ni exchange interaction parameters change as well (see Sec. 2.4 of the manuscript) and arrives at (all values in cm^{-1})

$$J^{(13)} = -1.694 \tag{S14}$$

$$\mathbf{D}^{(13)} = \begin{pmatrix} -2.861 & -0.248 & 0.547 \\ -0.248 & 3.789 & 2.684 \\ 0.547 & 2.864 & -0.928 \end{pmatrix} \tag{S15}$$

so these parameters have to be used in the spin Hamiltonian together with the single-centre $\mathbf{g}$ matrices and $\mathbf{D}$ tensors obtained in the preceding section. Note how the spin rotation re-shuffles the contribution to the exchange interaction between the isotropic and anisotropic parts. For example, the isotropic exchange coupling constant is now *anti*ferromagnetic, although the original and ,,rotated" parameters describe exactly the same physics.

The anisotropic exchange tensors $\mathbf{D}^{(13)}$, $\mathbf{D}^{(14)}$, $\mathbf{D}^{(15)}$ on one one hand, and $\mathbf{D}^{(23)}$, $\mathbf{D}^{(24)}$, $\mathbf{D}^{(25)}$ on the other, are related by symmetry. Fortunately, the spin rotation, especially the angle ϕ determining the rotation matrix (Eq. S13) is the same in all cases, such that we can give here the complete set of spin Hamiltonian parameters for the two-centre exchange interactions (all values are in cm^{-1})

$$\textbf{unscaled } J^{(\text{Os}_1\text{Ni}_3)} = J^{(\text{Os}_1\text{Ni}_4)} = J^{(\text{Os}_1\text{Ni}_4)} = -1.694 \tag{S16}$$

$$\textbf{unscaled } J^{(\text{Os}_2\text{Ni}_3)} = J^{(\text{Os}_2\text{Ni}_4)} = J^{(\text{Os}_2\text{Ni}_4)} = -1.668 \tag{S17}$$

$$\textbf{unscaled } \mathbf{D}^{(\mathrm{Os_1Ni_3})} = \begin{pmatrix} -2.861 & -0.248 & 0.547 \\ -0.248 & 3.789 & 2.684 \\ 0.547 & 2.864 & -0.928 \end{pmatrix} \tag{S18}$$

$$\textbf{unscaled } \mathbf{D}^{(\mathrm{Os_1Ni_4})} = \begin{pmatrix} 2.340 & -2.755 & 2.051 \\ -2.755 & -1.413 & -1.816 \\ 2.051 & -1.816 & -0.928 \end{pmatrix} \tag{S19}$$

$$\textbf{unscaled } \mathbf{D}^{(\mathrm{Os_1Ni_5})} = \begin{pmatrix} 1.912 & 3.003 & -2.598 \\ 3.003 & -0.984 & -0.868 \\ -2.598 & -0.868 & -0.928 \end{pmatrix} \tag{S20}$$

$$\textbf{unscaled } \mathbf{D}^{(\mathrm{Os_2Ni_3})} = \begin{pmatrix} -2.820 & 0.201 & -0.444 \\ 0.201 & 3.790 & 2.709 \\ -0.444 & 2.709 & -0.970 \end{pmatrix} \tag{S21}$$

$$\textbf{unscaled } \mathbf{D}^{(\mathrm{Os_2Ni_4})} = \begin{pmatrix} 1.963 & -2.963 & 2.568 \\ -2.963 & -0.994 & -0.969 \\ 2.568 & -0.969 & -0.970 \end{pmatrix} \tag{S22}$$

$$\textbf{unscaled } \mathbf{D}^{(\mathrm{Os_2Ni_5})} = \begin{pmatrix} 2.312 & 2.762 & -2.124 \\ 2.762 & -1.342 & -1.739 \\ -2.124 & -1.739 & -0.970 \end{pmatrix} \tag{S23}$$

To summarise, the following spin Hamiltonian parameters have been extracted from the CASOCI calculation:

- Os $\mathbf{g}^{(i)}$ matrices from Eqs. (S5, S6),
- Ni $\mathbf{g}^{(i)}$ matrices from Eqs. (S7–S9),
- Ni single-ion $\mathbf{D}^{(i)}$ tensors from Eqs. (S10–S12),
- isotropic Os-Ni $J^{(ij)}$ values from Eqs. (S16–S17),
- anisotropic Os-Ni $\mathbf{D}^{(ij)}$ tensors from Eqs. (S18–S23).

The Os-Ni exchange coupling parameters $J^{(ij)}$ and $\mathbf{D}^{(ij)}$ have been given the attribute ,,unscaled" in bold face since these are the parameters extracted ,,as is" from the CASOCI result. It turned out that when using them to reproduce experimental data, the exchange interactions have to be scaled by a factor of 2.5. These are the ,,scaled" results (Eqs. S24–S31) which result from the ,,unscaled" ones (Eqs. S16–S23) through multiplication with 2.5.

$$\textbf{scaled } J^{(\mathrm{Os_1Ni_3})} = J^{(\mathrm{Os_1Ni_4})} = J^{(\mathrm{Os_1Ni_4})} = -4.234 \tag{S24}$$

$$\textbf{scaled } J^{(\mathrm{Os_2Ni_3})} = J^{(\mathrm{Os_2Ni_4})} = J^{(\mathrm{Os_2Ni_4})} = -4.171 \tag{S25}$$

$$\textbf{scaled } \mathbf{D}^{(\mathrm{Os_1Ni_3})} = \begin{pmatrix} -7.151 & -0.619 & 1.367 \\ -0.619 & 9.471 & 6.711 \\ 1.367 & 6.711 & -2.319 \end{pmatrix} \tag{S26}$$

$$\textbf{scaled } \mathbf{D}^{(\mathrm{Os_1Ni_4})} = \begin{pmatrix} 5.851 & -6.888 & 5.128 \\ -6.888 & -3.532 & -4.539 \\ 5.128 & -4.539 & -2.319 \end{pmatrix} \tag{S27}$$

$$\textbf{scaled } \mathbf{D}^{(\mathrm{Os_1Ni_5})} = \begin{pmatrix} 4.779 & 7.507 & -6.495 \\ 7.507 & -2.460 & -2.171 \\ -6.495 & -2.171 & -2.319 \end{pmatrix} \tag{S28}$$

$$\textbf{scaled } \mathbf{D}^{(\mathrm{Os_2Ni_3})} = \begin{pmatrix} -7.051 & 0.503 & -1.111 \\ 0.503 & 9.475 & 6.772 \\ -1.111 & 6.772 & -2.424 \end{pmatrix} \tag{S29}$$

$$\textbf{scaled } \mathbf{D}^{(\mathrm{Os_2Ni_4})} = \begin{pmatrix} 4.908 & -7.407 & 6.420 \\ -7.407 & -2.484 & -2.424 \\ 6.420 & -2.424 & -2.424 \end{pmatrix} \tag{S30}$$

$$\textbf{scaled } \mathbf{D}^{(\mathrm{Os_2Ni_5})} = \begin{pmatrix} 5.779 & 6.904 & -5.309 \\ 6.904 & -3.355 & -4.348 \\ -5.309 & -4.348 & -2.424 \end{pmatrix} \tag{S31}$$

S1.4 Validating the spin Hamiltonian parameters

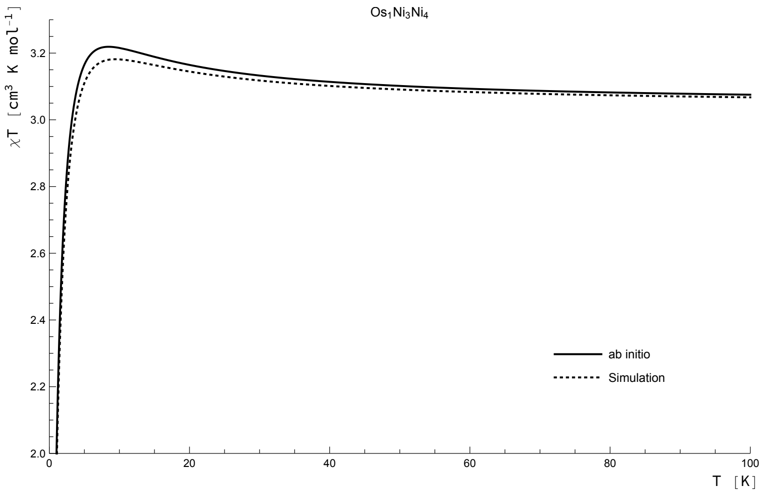

Figure S1: Temperature dependence of χT for the complex with Os(1), Ni(3) and Ni(4) ,,unmuted". ab initio: from CASOCI calculations, Simulation: from a spin Hamiltonian with the *unscaled* parameters given above.

The spin Hamiltonian parameters just given have been extracted from three sets of calculations, namely a) calculations where single Os atom was ,,unmuted" to

extract the g matrices of Os_1 and Os_2, b) calculations where a single Ni atom was ,,unmuted" to extract the g matrices and single-ion **D** tensors of Ni_3, Ni_4 and Ni_5, and c) calculations where one Os-Ni pair was ,,unmuted" to extract the isotropic exchange coupling constants and the anisotropic exchange tensors. The goal is, of course, to use these parameters to model the magnetic properties of the real complex with 5 active spin centres. Since it is not very difficult to perform CASOCI calculations with up to three ,,unmuted" centres, we now check whether results from a spin Hamiltonian reproduce magnetic properties calculated with CASOCI. In the spin Hamiltonian, we use the parameters given in the last section. Since we want to compare the results from spin Hamiltonian and CASOCI calculations, it is clear that we have to use the **unscaled** two-centre exchange parameters to perform this test. We consider cases with three ,,unmuted" centres, and there are three setups not equivalent by symmetry, namely $Os_1Ni_3Ni_4$, $Os_2Ni_3Ni_4$ and $Os_1Os_2Ni_3$ (the ,,unmuted" centres are denoted in the name). We made no comparison for the $Ni_3Ni_4Ni_5$ case since these three centres are all un-coupled in the spin Hamiltonian. We compare χT curves from CASOCI (solid lines) and the spin Hamiltonian (dotted lines) for temperatures from 0 to 100 K (beyond that, the curves are rather boring). These curves have been calculated for an external magnetic field of 0.5 Tesla. The low-T part of the curve will show sizeable differences if relative state energies deviate, while the high-T part is sensitive to the magnetic moments of the spin centres.

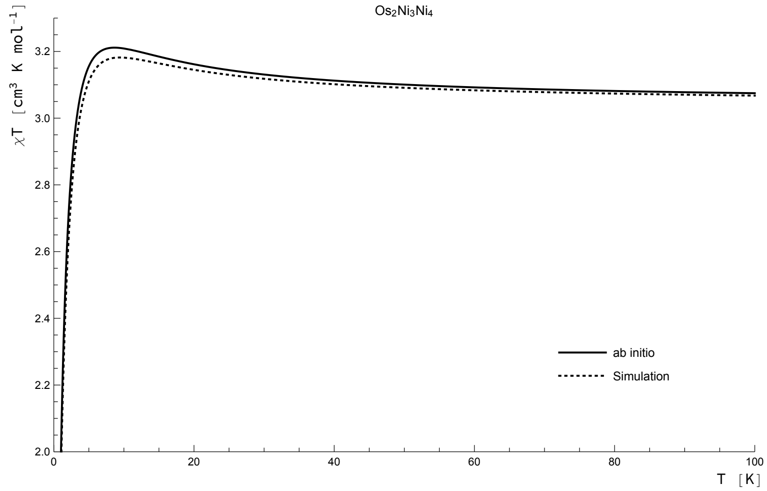

Figure S2: Temperature dependence of χT for the complex with Os(2), Ni(3) and Ni(4) ,,unmuted". ab initio: from CASOCI calculations, Simulation: from a spin Hamiltonian with the *unscaled* parameters given above.

Figures S1–S3 show the calculated χT curves for the three cases. For the overall shape of the curves, as well as the high-T limit, we find good agreement between the curves extracted from ab initio and spin Hamiltonian calculations.

The maximum susceptibility is found at very similar temperatures, while the peak susceptibility value is a little higher for the CASOCI than for the spin Hamiltonian calculations.

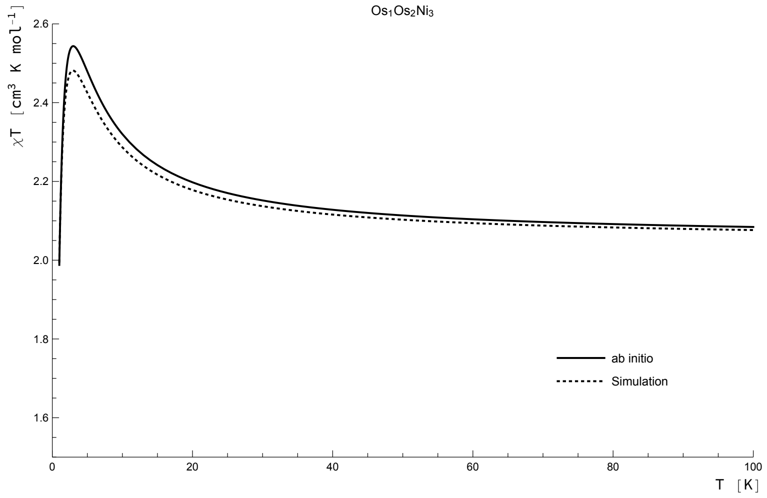

Figure S3: Temperature dependence of χT for the complex with Os(1), Os(2) and Ni(3) ,,unmuted”. ab initio: from CASOCI calculations, Simulation: from a spin Hamiltonian with the *unscaled* parameters given above.

S1.5 Scalar-relativistic isotropic exchange in the OsNi pairs

Table S2: Scalar-relativistic CASCI energy levels (in cm^{-1}, relative to the ground state) for two variants in which all centres except Os_1 and Ni_3 (second column) or all centres except Os_2 and Ni_3 (third column) are „muted". From the quartet-doublet splittings, the isotropic exchange coupling constants J_i are given for each of the three ligand-field split components.

Spin	Os_1Ni_3	Os_2Ni_3
$E(S = 3/2)$	0.00	0.00
$E(S = 1/2)$	4.73	4.67
J_1	3.15	3.11
$E(S = 3/2)$	400.49	401.27
$E(S = 1/2)$	417.37	417.41
J_2	11.25	10.76
$E(S = 3/2)$	432.18	433.53
$E(S = 1/2)$	434.48	436.06
J_3	1.53	1.69

Without spin-orbit coupling, the tree components of the $^2T_{2g}$ of the Os^{III} centre are split by the ligand field (by ~ 400 cm^{-1}). Each of these three doublets couples with the Ni^{II} centre when calculating an Os-Ni pair (that is, when muting all spin centres except one Os and one Ni centre). Because the coupling is weakly ferromagnetic in all cases, we get as a result three groups of states where in each group there is a quartet slightly below a doublet, and the energy difference amounts to $3/2J$, where J is the scalar-relativistic isotropic exchange-coupling constant for that Os-Ni pair. Anisotropic exchange can be attributed to the *differences* of the isotropic J values from the scalar relativistic calculations. Therefore, we list here the scalar-relativistic energy levels for the Os-Ni pairs (Table S2) together with the resulting scalar exchange coupling constants. The Os_1Ni_4 and Os_1Ni_5 pairs are symmetry equivalent to Os_1Ni_3, but the pairs involving Os_2 are slightly different. Note that the upper two components (at ~ 400 cm^{-1}) are degenerate in C_3 symmetry and split here because the diamagnetic substitution (two of the three equivalent Ni^{II} are replaced by Zn^{II}) destroys the C_3 symmetry axis.

S2 Effect of the Ni single-ion ZFS tensors

As a numerical experiment, a spin Hamiltonian calculation using the **g** and single-ion **D** tensors given above (Eqs. S5–S6,S7–S12) together with the **scaled** two-centre exchange coupling constants (Eqs. S24–S31) was compared with a calculation where the parameters were all the same except that the single-ion

D tensors at Ni (Eqs. S10–S12) were set to zero. Note that this does not correspond to switching off the spin-orbit interaction at the Ni centres since the Ni g values being larger than 2 (Eqs. S7–S9). This experiment was performed to demonstrate the interplay between the Os-Ni anisotropic exchange and the Ni single-ion zero field splitting.
The resulting χT curves for both cases is shown in Fig S4. It can be seen that the calculated χT curve *without* Ni single-ion **D** tensors (dashed line) differs little from the curve obtained by the full spin Hamiltonian (solid line).

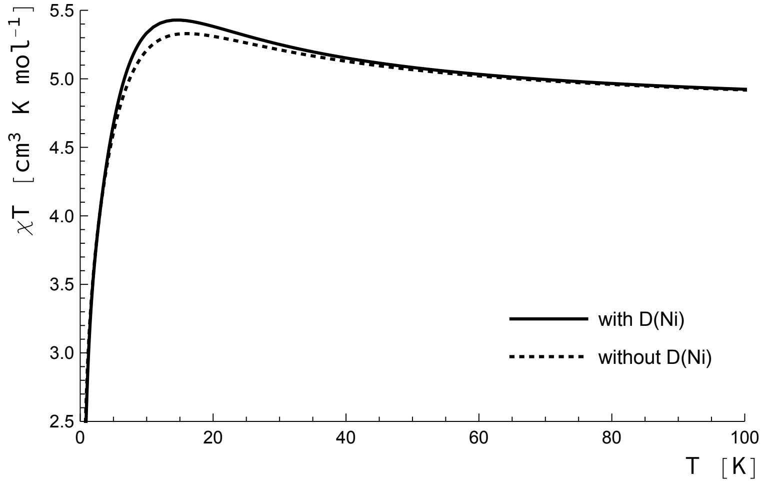

Figure S4: Temperature dependence of χT for the Os_2Ni_3 complex from spin Hamiltonian calculations using parameters extracted from CASOCI wave functions (solid line). Note that the **scaled** two-centre exchange interaction parameters have been used. The dashed line has been obtained by using the same set of parameters but setting the Ni single-ion **D** tensors to zero.

S3 ROHF Orbital optimisation

The restricted open-shell Hartree-Fock (ROHF) calculations are only used to optimise orbitals to be used in subsequent spin-orbit CAS-CI calculations. However, just taking the ground state configuration (a t_{2g}^5 configuration for Os and a $t_{2g}^6 e_g^2$ configuration for Ni) is not adequate to provide such orbitals. First, the e_g orbitals at Os are not occupied in the ROHF ground configuration and will therefore not be optimised, and second, the t_{2g} orbitals at Ni are fully occupied and will mix with all the other doubly occupied (ligand) orbitals. Therefore, we have to mix in excited configuration into the ROHF procedure. With ROHF (Roothaan-type) calculations, we can optimise the orbitals for an energy expression which is a weighted average of energy expectation values of an arbitrary number of Slater determinants. So for a calculation with a single un-muted Os atom, we take the three low-energy ($M_S = \frac{1}{2}$) determinants from the t_{2g}^5 configuration and the single low-energy ($M_S = \frac{5}{2}$) determinant from the excited $t_2g^4 e_g^2$ configuration, and give the former a total weight of 0.9 and the latter a weight of 0.1. This leads to an average occupation $t_{2g}^{4.8} e_g^{0.2}$ (on the average, 0.2 electrons are promoted). Likewise, for a calculation with a single un-muted Ni atom, we take the ground state determinant $t_{2g}^6 e_g^2$ with a weight of 0.8 and the six determinants from an excited $t_{2g}^5 e_g^3$ configuration with a summed weight of 0.2, leading to an average occupation of $t_{2g}^{5.8} e_g^{2.2}$ (again, 0.2 electrons promoted). This procedure takes care that we can decent optimised Os(e_g) orbitals, and Ni(t_{2g}) orbitals that do not mix with the ligand orbitals. So far, the orbitals could equally well have been obtained with a state-averaged CASSCF procedure, which is what one usually does if there are orbitals that are too weakly or too strongly occupied.

However the standard state-averaged CASSCF procedure cannot be used to optimised orbitals for systems with more than one metal centre ,,un-muted". Consider a case where we have one Os and one Ni centre. We want a size extensive orbitals optimisation procedure, that is, for virtually non-interacting metal centres we want the same optimised orbitals at each centre that we got from a calculation with only a single ,,un-muted" centre. In a state-averaged CASSCF calculation this requires that we include all states which a products of states with up to single excitation at any of the metal centre, in our case, states which are simultaneously ligand-field excited at the Os and at the Ni centres. Such two-centre excitations are most likely above single-centre excitations that we did no include in the one-centre case, so it is just not possible to include exactly those states in a state-averaged CASSCF calculation. With our ROHF orbital optimisation we can ensure size consistency. In the OsNi example, we include the three ground state determinants (Os t_{2g}^5)(Ni $t_{2g}^6 e_g^2$) with a summed weight of 0.72 (0.8 * 0.9), the single determinant from Os excitation (Os $t_{2g}^3 e_g^2$)(Ni $t_{2g}^6 e_g^2$) with a weight of 0.08, 18 determinants from Ni excitation (Os t_{2g}^5)(Ni $t_{2g}^5 e_g^3$) with a summed weight of 0.18, and finally six determinants that describe excitation at both centres (Os $t_{2g}^3 e_g^2$)(Ni $t_{2g}^5 e_g^3$) with a weight of 0.02. This way, the average occupation numbers match those of the single-centre calculations and the molecular orbitals at each centre feel the same effective field in all calcula-

tions, independent of whether other centres are muted or unmuted. Note that a large number of Slater determinants in the ROHF average does not affect the computational effort, the Roothaan a and b coefficients are calculated with the help of a MATHEMATICA document within seconds. The computational effort does, however, depend on the total number of open shells (two per un-muted centre).

Note that orbital optimisation cannot be done via standard Fock matrix diagonalisation, we must use Jacobi rotations instead. The reason is, that the optimised orbitals are necessarily localised. In a complex with, say, two unmuted Ni centres, Slater determinants with orbitals delocalised over both Ni centres will contain contributions from charge-transfer excitations (from one Ni centre to the other) which are high in energy. Therefore, we localise the ,,active" orbitals on the spin centres before starting the ROHF orbital optimisation.

All ROHF calculations were performed with two open shells (t_{2g}, e_g) per ,,unmuted" metal centre. The ROHF energy expression to be minimised is a weighted average of the energy expression of a selection of Slater determinants, and this set is chosen to be size consistent (see last section). Therefore, the resulting occupation numbers of each type of shell is the same no matter how many metal centres are ,,unmuted". For completeness, the occupation numbers (0 corresponds to unoccupied in all Slater determinants and 2 corresponds to doubly occupied in all Slater determinants) are given in Table S3.

Table S3: Occupation number of metal-centred open shell orbitals

Shell Type	occupation number
Os t_{2g}	$\frac{8}{5}$
Os e_g	$\frac{1}{10}$
Ni t_{2g}	$\frac{29}{15}$
Ni e_g	$\frac{11}{10}$

The Roothaan a and b parameters are the same for each shell pair of the same type. This is also the consequence of properly choosing the Slater determinants and weights. For two open shells located at the same centre the Roothaan parameters are given in Table S4. These values reflect the amount of ,,excited" Slater determinants in the energy expression that is necessary to optimise the Os e_g orbitals and not to mix the Ni t_{2g} orbitals with the closed-shell (ligand) ones.

Finally, Table S5 shows the Roothaan parameters for two shells located at different centres. Note that the a parameter is unity in all cases, which ensures correct long-range electrostatics.

Table S4: Roothaan parameters for two open shells located at the same centre

shell pair		a	b
Os(t_{2g})	Os(t_{2g})	$\frac{125}{128}$	$\frac{65}{64}$
Os(t_{2g})	Os(e_g)	$\frac{5}{8}$	$\frac{5}{4}$
Os(e_g)	Os(e_g)	10	20
Ni(t_{2g})	Ni(t_{2g})	$\frac{840}{841}$	$\frac{840}{841}$
Ni(t_{2g})	Ni(e_g)	$\frac{315}{319}$	$\frac{320}{319}$
Ni(e_g)	Ni(e_g)	$\frac{120}{121}$	$\frac{200}{121}$

Table S5: Roothaan parameters for two open shells located at different centres

shell pair		a	b
Os(t_{2g})	Os′(t_{2g})	1	$\frac{17}{16}$
Os(t_{2g})	Os′(e_g)	1	$\frac{5}{4}$
Os(e_g)	Os′(e_g)	1	2
Ni(t_{2g})	Ni′(t_{2g})	1	$\frac{842}{841}$
Ni(t_{2g})	Ni′(e_g)	1	$\frac{328}{319}$
Ni(e_g)	Ni′(e_g)	1	$\frac{202}{121}$
Os(t_{2g})	Ni(t_{2g})	1	$\frac{117}{116}$
Os(t_{2g})	Ni(e_g)	1	$\frac{53}{44}$
Os(e_g)	Ni(t_{2g})	1	$\frac{30}{29}$
Os(e_g)	Ni(e_g)	1	$\frac{20}{11}$

S4 Basis sets used for Ir, Os, Zn, Ni in TURBOMOLE format

S4.1 Ir basis

```
*
ir HiraoNakajimaContDKH
*
   10  s
   154077280000.           .52985434889846D-05
   16236210000.0           .18669372241760D-04
   2564766000.00           .53964567454775D-04
   536570140.000           .13354111122818D-03
   130788620.000           .31540817737409D-03
   35093707.0000           .70775323997332D-03
   10210191.0000           .15121800375813D-02
   3185316.70000           .31215996555672D-02
   1047978.80000           .63290740529048D-02
   361606.830000           .12499526786299D-01
   1  s
   130906.260000           1.00000000000
   1  s
   49287.4760000           1.00000000000
   1  s
   19392.2950000           1.00000000000
   1  s
   7935.98830000           1.00000000000
   1  s
   3324.94590000           1.00000000000
   1  s
   1423.71260000           1.00000000000
   1  s
   626.973540000           1.00000000000
   1  s
   252.284490000           1.00000000000
   1  s
   114.591080000           1.00000000000
   1  s
   46.2524100000           1.00000000000
   1  s
   24.1289590000           1.00000000000
   1  s
   9.73665030000           1.00000000000
   1  s
   4.90805110000           1.00000000000
```

```
 1  s
 1.48270940000           1.00000000000
 1  s
0.705139740000           1.00000000000
 1  s
0.136231700000           1.00000000000
 1  s
0.497745370000E-01       1.00000000000
 6  p
 53288951.0000           .12298199663910D-04
 7574870.60000           .40508205888343D-04
 1564127.20000           .11693219543957D-03
 399411.600000           .31690260302381D-03
 118743.750000           .84112094755503D-03
 39762.6140000           .22492739858891D-02
 1  p
 14630.8670000           1.00000000000
 1  p
 5839.36480000           1.00000000000
 1  p
 2503.75400000           1.00000000000
 1  p
 1142.88920000           1.00000000000
 1  p
 550.213670000           1.00000000000
 1  p
 276.307420000           1.00000000000
 1  p
 143.625350000           1.00000000000
 1  p
 76.3711450000           1.00000000000
 1  p
 41.4449130000           1.00000000000
 1  p
 22.9202410000           1.00000000000
 1  p
 12.5968150000           1.00000000000
 1  p
 6.81101120000           1.00000000000
 1  p
 3.67098460000           1.00000000000
 1  p
 1.92918850000           1.00000000000
 1  p
0.971374760000           1.00000000000
```

```
 1  p
0.463208960000           1.00000000000
 1  p
0.180762450000           1.00000000000
 1  p
0.070000000000           1.00000000000
 5  d
 17150.2900000           .12316892565929D-03
 4430.14010000           .87231387520276D-03
 1570.06630000           .47548755207752D-02
 652.558500000           .20947283018579D-01
 297.988380000           .72467288184584D-01
 1  d
 144.812270000           1.00000000000
 1  d
 73.0490890000           1.00000000000
 1  d
 37.6436000000           1.00000000000
 1  d
 19.4061760000           1.00000000000
 1  d
 9.75365430000           1.00000000000
 1  d
 4.78300410000           1.00000000000
 1  d
 2.22847090000           1.00000000000
 1  d
0.973650180000           1.00000000000
 1  d
0.393051120000           1.00000000000
 1  d
0.142649630000           1.00000000000
 1  d
0.050000000000           1.00000000000
 6  f
 832.740200000           .36881801242663D-03
 315.336140000           .26708168931413D-02
 139.466400000           .14551477385976D-01
 66.5635910000           .53103638786675D-01
 33.4419240000           .13822590510999
 17.2622820000           .25817331116659
 1  f
 8.82478860000           1.00000000000
 1  f
 4.40243210000           1.00000000000
```

```
   1  f
   2.07386950000            1.00000000000
   1  f
  0.858105900000            1.00000000000
   1 f
  0.300000000000            1.00000000000
   1 f
  0.100000000000            1.00000000000
*
```

S4.2 Os basis

```
*
os HiraoNakajimaContDKH
*
   10 s
   165337300000.           .47601492134017D-05
   16450041000.0           .17341785127585D-04
   2573461700.00           .51067501498565D-04
   516023820.000           .13157111658808D-03
   123867700.000           .30587122733342D-03
   33755743.0000           .67522277887954D-03
   9914548.30000           .14556513275562D-02
   3096957.40000           .30022591465593D-02
   1028553.00000           .60373324380625D-02
   357428.370000           .11977156619059D-01
   1  s
   129857.630000            1.00000000000
   1  s
   49115.8040000            1.00000000000
   1  s
   19341.9820000            1.00000000000
   1  s
   7901.61920000            1.00000000000
   1  s
   3302.17770000            1.00000000000
   1  s
   1410.61570000            1.00000000000
   1  s
   618.649590000            1.00000000000
   1  s
   245.673790000            1.00000000000
   1  s
   111.222190000            1.00000000000
```

```
 1  s
 44.7165320000          1.00000000000
 1  s
 23.2977120000          1.00000000000
 1  s
 9.32122460000          1.00000000000
 1  s
 4.67223020000          1.00000000000
 1  s
 1.35659830000          1.00000000000
 1  s
0.641380410000          1.00000000000
 1  s
0.130636250000          1.00000000000
 1  s
0.475413800000E-01      1.00000000000
 6  p
 54369194.0000          .11346833741493D-04
 7742180.70000          .37360421113163D-04
 1594791.70000          .10857832575540D-03
 403722.040000          .29775759270181D-03
 118751.460000          .79948277423002D-03
 39489.1760000          .21448694046463D-02
 1  p
 14546.8550000          1.00000000000
 1  p
 5828.75060000          1.00000000000
 1  p
 2504.39160000          1.00000000000
 1  p
 1143.22450000          1.00000000000
 1  p
 550.290170000          1.00000000000
 1  p
 276.830610000          1.00000000000
 1  p
 144.269160000          1.00000000000
 1  p
 76.7954050000          1.00000000000
 1  p
 41.5872880000          1.00000000000
 1  p
 23.0131500000          1.00000000000
 1  p
 12.6656530000          1.00000000000
```

```
 1  p
 6.84126480000          1.00000000000
 1  p
 3.68641770000          1.00000000000
 1  p
 1.90041960000          1.00000000000
 1  p
0.967687550000          1.00000000000
 1  p
0.473394060000          1.00000000000
 1  p
0.197173990000          1.00000000000
 1  p
0.070000000000          1.00000000000
 5  d
 17143.7430000          .11480231342218D-03
 4428.13310000          .81499186753638D-03
 1569.10910000          .44591370511315D-02
 652.267030000          .19727374196040D-01
 297.996420000          .68750822731752D-01
 1  d
 144.797610000          1.00000000000
 1  d
 73.0181900000          1.00000000000
 1  d
 37.6001350000          1.00000000000
 1  d
 19.4136920000          1.00000000000
 1  d
 9.75582590000          1.00000000000
 1  d
 4.78212480000          1.00000000000
 1  d
 2.24589910000          1.00000000000
 1  d
0.972261570000          1.00000000000
 1  d
0.391958330000          1.00000000000
 1  d
0.141080790000          1.00000000000
 1  d
0.050000000000          1.00000000000
```

```
   6  f
   1032.78560000           .23489140574625D-03
   328.202970000           .24977071378161D-02
   136.868440000           .14034879126661D-01
   65.5969990000           .49233215696589D-01
   33.3710140000           .12772632552047
   17.2974010000           .24729419508937
   1  f
   8.78053240000           1.00000000000
   1  f
   4.30093370000           1.00000000000
   1  f
   1.98241560000           1.00000000000
   1  f
  0.807655390000           1.00000000000
   1 f
  0.300000000000           1.00000000000
   1 f
  0.100000000000           1.00000000000
*
```

S4.3 Zn basis

```
*
zn HiraoNakajimaContDKH
*
   7  s
   70817880.0000         0.31214947855236D-04
   7572294.00000         0.12019552810480D-03
   1323068.00000         0.36860596242984D-03
   302505.700000         0.10086258397525D-02
   82854.2800000         0.26080247355833D-02
   25950.9000000         0.66412776131427D-02
   8984.71000000         0.17120522783515D-01
   1  s
   3368.58800000           1.00000000000
   1  s
   1348.75800000           1.00000000000
   1  s
   567.243900000           1.00000000000
   1  s
   248.306300000           1.00000000000
   1  s
   111.905500000           1.00000000000
   1  s
   50.4261700000           1.00000000000
```

```
 1  s
 23.4282000000          1.00000000000
 1  s
 11.0912600000          1.00000000000
 1  s
 4.89705600000          1.00000000000
 1  s
 2.25380200000          1.00000000000
 1  s
0.961268000000          1.00000000000
 1  s
0.155777000000          1.00000000000
 1  s
0.531356600000E-01      1.00000000000
 5  p
 57518.0100000          0.66722099374679D-04
 9362.77200000          0.40596158084390D-03
 2418.08400000          0.21837344776408D-02
 800.053500000          0.10002410956356D-01
 310.058400000          0.37186266977416D-01
 1  p
 133.372500000          1.00000000000
 1  p
 61.7014800000          1.00000000000
 1  p
 29.9587300000          1.00000000000
 1  p
 15.1082500000          1.00000000000
 1  p
 7.75174700000          1.00000000000
 1  p
 4.08510600000          1.00000000000
 1  p
 2.28201900000          1.00000000000
 1  p
 1.37071600000          1.00000000000
 1  p
0.747676500000          1.00000000000
 1  p
0.318787700000          1.00000000000
 1  p
0.120000000000          1.00000000000
```

```
   4  d
   299.803200000            0.11199688340410D-02
   91.7951700000            0.87259593110803D-02
   35.6373700000            0.38915599896750D-01
   15.2162700000            0.11802478075951D+00
   1  d
   6.85369200000            1.00000000000
   1  d
   3.09721700000            1.00000000000
   1  d
   1.35784900000            1.00000000000
   1  d
  0.565239400000            1.00000000000
   1  d
  0.212510500000            1.00000000000
   1  d
  0.090000000000            1.00000000000
   1  f
   8.02000000000            1.00000000000
   1  f
   2.61400000000            1.00000000000
   1  f
  0.836000000000            1.00000000000
   1  f
  0.267300000000            1.00000000000
*
```

S4.4 Ni basis

```
*
ni HiraoNakajimaContDKH
*
   7  s
   68573180.0000            .26055643675704D-04
   7371292.00000            .99819012378407D-04
   1301782.00000            .30547548997974D-03
   298708.500000            .83686893041186D-03
   82337.3500000            .21605489449715D-02
   25855.3700000            .55376817003918D-02
   8964.77700000            .14323942459839D-01
   1  s
   3364.19400000            1.00000000000
   1  s
   1342.58400000            1.00000000000
   1  s
   564.568700000            1.00000000000
```

```
 1  s
 248.272400000          1.00000000000
 1  s
 113.303100000          1.00000000000
 1  s
 53.0561600000          1.00000000000
 1  s
 22.8113400000          1.00000000000
 1  s
 10.6779300000          1.00000000000
 1  s
 4.88033300000          1.00000000000
 1  s
 2.10786900000          1.00000000000
 1  s
0.869886900000          1.00000000000
 1  s
0.136735900000          1.00000000000
 1  s
0.478924000000E-01      1.00000000000
 5  p
 51568.3300000          .56134216570771D-04
 8804.86300000          .32578558020234D-03
 2375.40100000          .16749138777984D-02
 803.176400000          .76613485971063D-02
 311.538300000          .29280896812372D-01
 1  p
 133.462700000          1.00000000000
 1  p
 61.7126800000          1.00000000000
 1  p
 30.0271800000          1.00000000000
 1  p
 15.1472300000          1.00000000000
 1  p
 7.82462400000          1.00000000000
 1  p
 4.14289900000          1.00000000000
 1  p
 2.31968400000          1.00000000000
 1  p
 1.38149300000          1.00000000000
 1  p
0.731439500000          1.00000000000
 1  p
0.313133300000          1.00000000000
```

```
  1  p
 0.100000000000           1.00000000000
  4  d
  283.451500000           .90938100434331D-03
  80.7854700000           .82057246947639D-02
  30.1810000000           .38200673317335D-01
  12.6222300000           .11719098500239
  1  d
  5.62074200000           1.00000000000
  1  d
  2.52428700000           1.00000000000
  1  d
  1.10484700000           1.00000000000
  1  d
 0.462363400000           1.00000000000
  1  d
 0.177013000000           1.00000000000
  1  d
 0.070000000000           1.00000000000
  1  f
  6.75800000000           1.00000000000
  1  f
  2.17400000000           1.00000000000
  1  f
 0.681000000000           1.00000000000
  1  f
 0.213000000000           1.00000000000
*
```

S5 Molecular geometry of the neutral „Os_2Ni_3" complex

We give the cartesian coordinates (in Angstrom) of the pentanuclear complex in C_3 symmetry. The molecule-fixed coordinate system has been chosen such that Os(1) and Os(2) are on the positive and negative z axis, while Ni(3) is on the positive x axis. The neutral molecule contains 233 atoms.

```
Os(  1)      0.000000  0.000000  3.320534
Os(  2)      0.000000  0.000000 -3.316841
Ni(  3)      3.891722  0.000000  0.000000
Ni(  4)     -1.945861 -3.370330  0.000000
Ni(  5)     -1.945861  3.370330  0.000000
N (  6)      4.298034  2.072417 -0.702712
N (  7)      5.485775  0.850578  1.382366
N (  8)      5.492474 -0.835299 -1.378025
N (  9)      4.322936 -2.065317  0.711424
N ( 10)      1.844031 -1.980697  4.998477
N ( 11)      2.531859  0.375162  1.470097
N ( 12)      1.859002  1.962658 -4.999098
N ( 13)      2.531671 -0.389256 -1.468110
C ( 14)      3.717180  2.654542 -1.731267
H ( 15)      3.033949  2.042958 -2.313671
C ( 16)      3.935317  3.992769 -2.109239
C ( 17)      3.217911  4.526264 -3.309785
H ( 18)      3.910972  5.000923 -4.009176
H ( 19)      2.697868  3.730077 -3.847416
H ( 20)      2.478826  5.280402 -3.019637
C ( 21)      4.803640  4.751073 -1.341430
C ( 22)      5.085178  6.184463 -1.666044
H ( 23)      6.121050  6.316635 -1.994251
H ( 24)      4.434386  6.549606 -2.456860
H ( 25)      4.944288  6.822848 -0.790364
C ( 26)      5.432767  4.140637 -0.229395
C ( 27)      6.335523  4.834203  0.631178
H ( 28)      6.570915  5.870148  0.421722
C ( 29)      6.899873  4.231332  1.706185
H ( 30)      7.569047  4.797335  2.341988
C ( 31)      6.629624  2.866785  2.020761
C ( 32)      7.173490  2.214413  3.153423
C ( 33)      8.068708  2.962852  4.089547
H ( 34)      7.541089  3.808065  4.540742
H ( 35)      8.430664  2.329591  4.896184
H ( 36)      8.937928  3.368158  3.564267
C ( 37)      6.837521  0.888856  3.370522
C ( 38)      7.313356  0.100731  4.551845
```

H	(39)	6.984477	0.551845	5.491396
H	(40)	6.912514	-0.912717	4.521272
H	(41)	8.404056	0.030072	4.582843
C	(42)	5.995066	0.263569	2.438455
H	(43)	5.719745	-0.775868	2.588140
C	(44)	5.772024	2.143729	1.171985
C	(45)	5.151187	2.791913	0.046486
C	(46)	5.991553	-0.246011	-2.437711
H	(47)	5.693763	0.785824	-2.596758
C	(48)	6.850373	-0.860692	-3.361960
C	(49)	7.312402	-0.072286	-4.548570
H	(50)	7.000954	-0.540993	-5.485388
H	(51)	6.885216	0.930624	-4.530469
H	(52)	8.401116	0.026290	-4.574090
C	(53)	7.215391	-2.176445	-3.132057
C	(54)	8.131237	-2.912301	-4.058165
H	(55)	9.008168	-3.291486	-3.526195
H	(56)	7.625707	-3.774353	-4.502720
H	(57)	8.480701	-2.278303	-4.869735
C	(58)	6.682032	-2.831299	-1.995778
C	(59)	6.980993	-4.186877	-1.668321
H	(60)	7.665350	-4.743275	-2.296420
C	(61)	6.425372	-4.793179	-0.590619
H	(62)	6.682798	-5.821872	-0.371600
C	(63)	5.503482	-4.112731	0.260177
C	(64)	4.881996	-4.727227	1.374354
C	(65)	5.193687	-6.150963	1.713764
H	(66)	4.541449	-6.525762	2.498867
H	(67)	5.081004	-6.799746	0.841739
H	(68)	6.227890	-6.254973	2.057240
C	(69)	3.993234	-3.981503	2.131298
C	(70)	3.280595	-4.520458	3.332313
H	(71)	3.979553	-4.975701	4.038834
H	(72)	2.741249	-3.731278	3.861400
H	(73)	2.558805	-5.291911	3.044059
C	(74)	3.748992	-2.651148	1.741667
H	(75)	3.050653	-2.048923	2.315910
C	(76)	5.194281	-2.772648	-0.028031
C	(77)	5.805745	-2.120219	-1.156183
C	(78)	1.137892	-1.246429	4.447260
C	(79)	1.610663	0.283034	2.164171
C	(80)	1.147093	1.236016	-4.445202
C	(81)	1.609787	-0.293239	-2.160900
N	(82)	-0.354251	-4.758415	-0.702712
N	(83)	-3.943783	2.685998	-0.702712
N	(84)	-2.006265	-5.176110	1.382366

N (85)	-3.479510	4.325532	1.382366
N (86)	-3.469627	-4.338973	-1.378025
N (87)	-2.022847	5.174272	-1.378025
N (88)	-3.950085	-2.711114	0.711424
N (89)	-0.372851	4.776431	0.711424
N (90)	-2.637349	-0.606629	4.998477
N (91)	0.793319	2.587326	4.998477
N (92)	-0.941029	-2.380235	1.470097
N (93)	-1.590829	2.005073	1.470097
N (94)	0.770210	-2.591272	-4.999098
N (95)	-2.629212	0.628614	-4.999098
N (96)	-1.602941	-1.997864	-1.468110
N (97)	-0.928730	2.387120	-1.468110
C (98)	0.440311	-4.546444	-1.731267
C (99)	-4.157491	1.891902	-1.731267
H (100)	0.252279	-3.648956	-2.313671
H (101)	-3.286228	1.605998	-2.313671
C (102)	1.490180	-5.404469	-2.109239
C (103)	-5.425498	1.411701	-2.109239
C (104)	2.310904	-5.049925	-3.309785
C (105)	-5.528815	0.523661	-3.309785
H (106)	2.375440	-5.887463	-4.009176
H (107)	-6.286413	0.886540	-4.009176
H (108)	1.881407	-4.201461	-3.847416
H (109)	-4.579276	0.471384	-3.847416
H (110)	3.333549	-4.786927	-3.019637
H (111)	-5.812375	-0.493475	-3.019637
C (112)	1.712730	-6.535611	-1.341430
C (113)	-6.516370	1.784538	-1.341430
C (114)	2.813313	-7.496125	-1.666044
C (115)	-7.898491	1.311662	-1.666044
H (116)	2.409842	-8.459303	-1.994251
H (117)	-8.530892	2.142667	-1.994251
H (118)	3.454932	-7.115094	-2.456860
H (119)	-7.889318	0.565488	-2.456860
H (120)	3.436616	-7.693303	-0.790364
H (121)	-8.380904	0.870455	-0.790364
C (122)	0.869513	-6.775233	-0.229395
C (123)	-6.302281	2.634596	-0.229395
C (124)	1.018781	-7.903825	0.631178
C (125)	-7.354304	3.069622	0.631178
H (126)	1.798240	-8.625654	0.421722
H (127)	-8.369155	2.755505	0.421722
C (128)	0.214504	-8.091132	1.706185
C (129)	-7.114378	3.859800	1.706185
H (130)	0.370091	-8.953654	2.341988

H	(131)	-7.939138	4.156319	2.341988
C	(132)	-0.832104	-7.174816	2.020761
C	(133)	-5.797521	4.308031	2.020761
C	(134)	-1.669007	-7.319631	3.153423
C	(135)	-5.504483	5.105218	3.153423
C	(136)	-1.468449	-8.469132	4.089547
C	(137)	-6.600259	5.506280	4.089547
H	(138)	-0.472663	-8.434807	4.540742
H	(139)	-7.068426	4.626742	4.540742
H	(140)	-2.197847	-8.465965	4.896184
H	(141)	-6.232817	6.136373	4.896184
H	(142)	-1.552053	-9.424552	3.564267
H	(143)	-7.385875	6.056394	3.564267
C	(144)	-2.648988	-6.365895	3.370522
C	(145)	-4.188533	5.477039	3.370522
C	(146)	-3.569442	-6.383918	4.551845
C	(147)	-3.743914	6.283187	4.551845
H	(148)	-3.014327	-6.324657	5.491396
H	(149)	-3.970150	5.772813	5.491396
H	(150)	-4.246693	-5.530054	4.521272
H	(151)	-2.665821	6.442771	4.521272
H	(152)	-4.175984	-7.293162	4.582843
H	(153)	-4.228071	7.263089	4.582843
C	(154)	-2.769275	-5.323664	2.438455
C	(155)	-3.225791	5.060095	2.438455
H	(156)	-3.531794	-4.565510	2.588140
H	(157)	-2.187951	5.341378	2.588140
C	(158)	-1.029488	-6.070585	1.171985
C	(159)	-4.742536	3.926855	1.171985
C	(160)	-0.157726	-5.857015	0.046486
C	(161)	-4.993461	3.065103	0.046486
C	(162)	-3.208829	-5.065832	-2.437711
C	(163)	-2.782725	5.311843	-2.437711
H	(164)	-2.166338	-5.323855	-2.596758
H	(165)	-3.527425	4.538031	-2.596758
C	(166)	-4.170567	-5.502251	-3.361960
C	(167)	-2.679806	6.362942	-3.361960
C	(168)	-3.718803	-6.296583	-4.548570
C	(169)	-3.593599	6.368869	-4.548570
H	(170)	-3.968991	-5.792508	-5.485388
H	(171)	-3.031963	6.333501	-5.485388
H	(172)	-2.636664	-6.428084	-4.530469
H	(173)	-4.248552	5.497460	-4.530469
H	(174)	-4.177790	-7.288724	-4.574090
H	(175)	-4.223325	7.262435	-4.574090
C	(176)	-5.492552	-5.160489	-3.132057

C (177)	-1.722839	7.336934	-3.132057
C (178)	-6.587745	-5.585707	-4.058165
C (179)	-1.543492	8.498009	-4.058165
H (180)	-7.354595	-6.155559	-3.526195
H (181)	-1.653573	9.447045	-3.526195
H (182)	-7.081539	-4.716879	-4.502720
H (183)	-0.544168	8.491232	-4.502720
H (184)	-6.213419	-6.205351	-4.869735
H (185)	-2.267282	8.483654	-4.869735
C (186)	-5.792993	-4.371160	-1.995778
C (187)	-0.889039	7.202459	-1.995778
C (188)	-7.116438	-3.952279	-1.668321
C (189)	0.135445	8.139156	-1.668321
H (190)	-7.940472	-4.266751	-2.296420
H (191)	0.275121	9.010026	-2.296420
C (192)	-7.363701	-3.167946	-0.590619
C (193)	0.938328	7.961125	-0.590619
H (194)	-8.383288	-2.876536	-0.371600
H (195)	1.700490	8.698408	-0.371600
C (196)	-6.313471	-2.709790	0.260177
C (197)	0.809989	6.822521	0.260177
C (198)	-6.534896	-1.864319	1.374354
C (199)	1.652901	6.591546	1.374354
C (200)	-7.923734	-1.422383	1.713764
C (201)	2.730047	7.573346	1.713764
H (202)	-7.922200	-0.670130	2.498867
H (203)	3.380751	7.195892	2.498867
H (204)	-8.429255	-1.000406	0.841739
H (205)	3.348251	7.800152	0.841739
H (206)	-8.530910	-2.266025	2.057240
H (207)	2.303020	8.520997	2.057240
C (208)	-5.444700	-1.467491	2.131298
C (209)	1.451466	5.448994	2.131298
C (210)	-5.555129	-0.580850	3.332313
C (211)	2.274534	5.101308	3.332313
H (212)	-6.298860	-0.958544	4.038834
H (213)	2.319307	5.934245	4.038834
H (214)	-4.602006	-0.508352	3.861400
H (215)	1.860757	4.239630	3.861400
H (216)	-5.862332	0.429966	3.044059
H (217)	3.303527	4.861946	3.044059
C (218)	-4.170457	-1.921148	1.741667
C (219)	0.421466	4.572296	1.741667
H (220)	-3.299746	-1.617481	2.315910
H (221)	0.249093	3.666404	2.315910
C (222)	-4.998324	-3.112055	-0.028031

C (223)	-0.195957	5.884703	-0.028031
C (224)	-4.739036	-3.967813	-1.156183
C (225)	-1.066709	6.088032	-1.156183
C (226)	-1.648385	-0.362229	4.447260
C (227)	0.510493	1.608658	4.447260
C (228)	-0.560217	-1.536392	2.164171
C (229)	-1.050446	1.253358	2.164171
C (230)	0.496875	-1.611419	-4.445202
C (231)	-1.643967	0.375403	-4.445202
C (232)	-1.058846	-1.247497	-2.160900
C (233)	-0.550941	1.540736	-2.160900

S6 Restricted open shell Hartree-Fock energies

Here we document the ROHF energies obtained for the completely ,,muted" complex (all Os replaced by Ir, all Ni replaced by Zn) as well as some complexes with 1, 2, or 3 metal centres ,,unmuted". Note that our size-consistent definition of the ROHF energy expression leads to a fairly constant energy increment for each Ir→Os and Zn→Ni substitution. The partially ,,unmuted" complexes are denoted by listing the ,,unmuted" atoms.

Species	Energy [Hartree]
,,completely muted"	-46513.015768
Os(1)	-45938.308666
Os(2)	-45938.308824
Ni(3)	-46237.798047
Ni(4)	-46237.798155
Ni(5)	-46237.798264
Ni(3)Ni(4)	-45962.579985
Os(1)Ni(3)	-45663.090909
Os(1)Ni(4)	-45663.090829
Os(1)Ni(5)	-45663.090866
Os(2)Ni(3)	-45663.091103
Os(2)Ni(4)	-45663.091060
Os(2)Ni(5)	-45663.091267
Os(1)Ni(3)Ni(4)	-45387.873164
Os(1)Ni(3)Ni(5)	-45387.873189
Os(1)Ni(4)Ni(5)	-45387.873109
Os(2)Ni(3)Ni(4)	-45387.873363
Os(2)Ni(3)Ni(5)	-45387.873515
Os(2)Ni(4)Ni(5)	-45387.873289
Os(1)Os(2)Ni(3)	-45088.384041
Os(1)Os(2)Ni(4)	-45088.383893
Os(1)Os(2)Ni(5)	-45088.383980
